# Bibliometric analysis of childhood adversity and anxiety disorders: Trends, hotspots and international collaboration

**DOI:** 10.1017/gmh.2026.10235

**Published:** 2026-06-08

**Authors:** Nairong Ruan, Liuyin Jin, Yaping Chen, Feijie Zheng

**Affiliations:** 1 Ningbo Psychiatric Hospital, China; 2 The Second People’s Hospital of Lishui, Lishui, Zhejiang, China; 3 Ningbo Kangning Hospital, China

**Keywords:** childhood adversity, adverse childhood experiences, anxiety disorders, bibliometric analysis, thematic evolution

## Abstract

Anxiety disorders are highly prevalent mental health conditions and are closely associated with early-life adversity. Although research on childhood adversity and anxiety disorders has expanded rapidly, the overall knowledge structure and emerging trends remain unclear. This bibliometric study systematically searched English-language publications from Web of Science Core Collection, Scopus and PubMed. Bibliometrix was used to analyze annual publication trends, countries, institutions, authors and journals. VOSviewer was applied to construct keyword co-occurrence and institutional collaboration networks, and CiteSpace was used to visualize thematic evolution and keyword bursts. A total of 4,481 eligible publications were included. The annual number of publications increased steadily since 2000, with marked acceleration after 2010. Research output and influence were mainly concentrated in the United States and Western Europe. High-frequency keywords included anxiety disorders, depression, childhood abuse, adverse childhood experiences and post-traumatic stress responses. Keyword analyses indicated a thematic shift from specific trauma types and diagnostic categories toward cumulative adversity, cross-diagnostic risk factors, emotional regulation, psychological resilience and symptom networks. This field has evolved from descriptive correlational studies to an interdisciplinary area integrating multilayered and mechanistic perspectives. Future studies should strengthen longitudinal designs, cross-cultural samples and multimodal data integration.

## Graphical abstract

**Figure:**
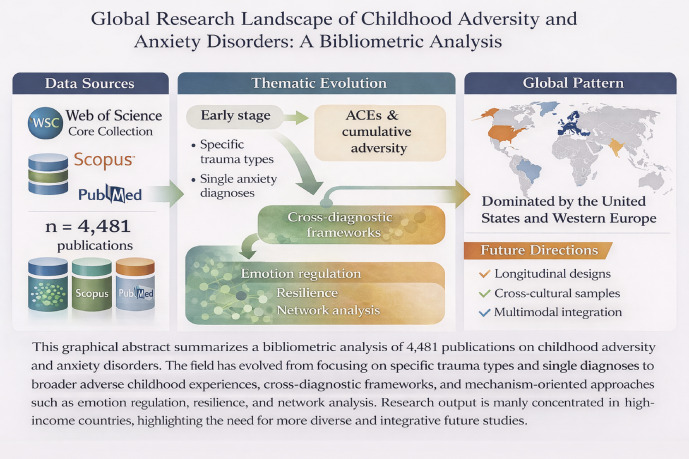

## Impact statement

Childhood adversity is a widespread and potentially preventable exposure with long-term implications for mental health, yet the evidence linking it to anxiety disorders has become increasingly complex and fragmented. By mapping the intellectual structure, international collaboration patterns and thematic evolution of this field, the present study provides a clearer foundation for researchers, clinicians and policymakers to understand where the evidence is concentrated and where important gaps remain. Rather than focusing only on individual trauma types or specific diagnostic categories, this work highlights a broader shift toward cumulative adversity, cross-diagnostic vulnerability and mechanism-oriented research. These insights may help guide future funding priorities, encourage more equitable global collaboration and support the development of earlier, more targeted prevention strategies for anxiety-related outcomes across diverse populations.

## Introduction

Anxiety is a natural response of humans when facing stress or danger. However, when this emotional reaction becomes too intense, frequent or prolonged, it may develop into an anxiety disorder (anxiety disorders), affecting ~1 in 10 people globally (Dean, 2016). The characteristics of anxiety disorders include worry, social and performance fear, panic attacks triggered unexpectedly, anticipatory anxiety and avoidance behaviors, with the prevalence continuously rising. Among these, the lifetime prevalence of generalized anxiety disorder is 6.2%, social anxiety disorder is 13% and panic disorder is 5.2% (whether or not accompanied by agoraphobia) (Szuhany and Simon, 2022). Globally, about 3.8% of the population, or ~285 million people, are affected (Disease et al., 2018).

In recent years, researchers have increasingly focused on the profound impact of early life experiences on mental health. Childhood adversity (CA) refers to various negative events experienced by individuals before the age of 18 years, including emotional neglect, physical or sexual abuse, domestic violence, parental separation, poverty and more (Kessler et al., 1997; Turk and Acet, 2026). It should be noted that CA, “early-life adversity” (ELA) and “adverse childhood experiences” (ACEs) are often used interchangeably in the literature, although their conceptual scopes are not entirely identical. In general, “early-life adversity” represents a broader construct, referring to adverse exposures occurring during early developmental periods, whereas “childhood adversity” more specifically denotes unfavorable experiences during childhood. In contrast, “ACEs” typically correspond to a more narrowly defined set of adverse experiences within the classical ACE framework (Remmers et al., 2024). Given that the present study focuses on the overall association between adverse experiences in childhood and early life and anxiety, “childhood adversity” is adopted as the primary umbrella term throughout this study. Meanwhile, related and conceptually overlapping terms were incorporated into the search strategy to ensure comprehensive coverage of the relevant literature. These early adverse experiences can not only have long-term effects on neurodevelopment but can also lead to widespread dysregulation of physiological systems due to the prolonged accumulation of psychosocial stress. Allostatic load (AL), a comprehensive indicator reflecting multisystem dysfunction, is considered capable of capturing the cumulative biological effects of chronic stress and has been proposed as a potential biological pathway linking childhood adversity to various mental health outcomes (Abrahamyan et al., 2026). In this framework, childhood adversity may increase vulnerability to anxiety disorders by promoting dysregulation of stress regulation systems. Furthermore, existing studies have shown a significant correlation between childhood adversity and the risk of anxiety disorders (Dalechek et al., 2024).

To gain a more comprehensive and systematic understanding of the development and trends in this field, bibliometric analysis has been widely applied in interdisciplinary areas such as psychology and public health in recent years. This method quantitatively analyzes scientific literature data through statistical analysis of indicators such as publication volume, citation frequency, key authors, research institutions, country distribution and keyword co-occurrence networks. It can reveal the evolutionary process, research hotspots and emerging trends in a particular field. Compared to traditional literature reviews, bibliometric analysis offers the advantages of high visualization, strong data objectivity and broad coverage. This study aims to use bibliometric methods to systematically analyze the international development status and academic structure of research on childhood adversity and anxiety relationships (Li et al., 2025). It should be noted that previous bibliometric studies have examined the overall developmental trends of research on broad ACEs, while some have also focused on specific mechanistic themes related to childhood trauma, such as epigenetics. These studies have indicated that mental health represents one of the major outcome domains in ACEs research, and anxiety has also been identified as a related topic within mechanistic studies of childhood trauma (Struck et al., 2021). However, these studies either approached the field from a broader perspective of ACEs or trauma research, encompassing multiple mental and physical health outcomes, and therefore did not specifically analyze anxiety as a distinct outcome domain (Chen et al., 2024; Sood et al., 2024; Sindhura and J, 2025) or they focused on a particular mechanistic branch, which remains insufficient to comprehensively capture the knowledge base, academic collaboration patterns, thematic clusters and emerging trends of research on the relationship between childhood adversity and anxiety (Tran et al., 2018; Ding and Lei, 2024). Therefore, the specific gap that the present study seeks to address is the lack of a systematic bibliometric analysis that specifically focuses on research concerning the relationship between childhood adversity and anxiety. Compared with previous studies, the present work does not aim to provide another broad overview of ACEs, trauma or mental health research; rather, it conceptualizes the association between childhood adversity and anxiety as a relatively independent knowledge domain. Specifically, this study aims to address the following questions: how research output in this field has evolved over time; which countries, institutions and authors constitute the core contributing forces; which types of adversity, anxiety phenotypes, population characteristics and mechanistic pathways represent the major research hotspots; whether the field is shifting from descriptive association toward mechanistic integration and precision-oriented intervention; and which thematic, population-based and regional gaps still remain. By addressing these questions, this study aims to provide more structured and evidence-based insights to support future theoretical integration, research design and the optimization of early intervention strategies.

## Methods

### Data sources and literature retrieval

**Figure 1.**
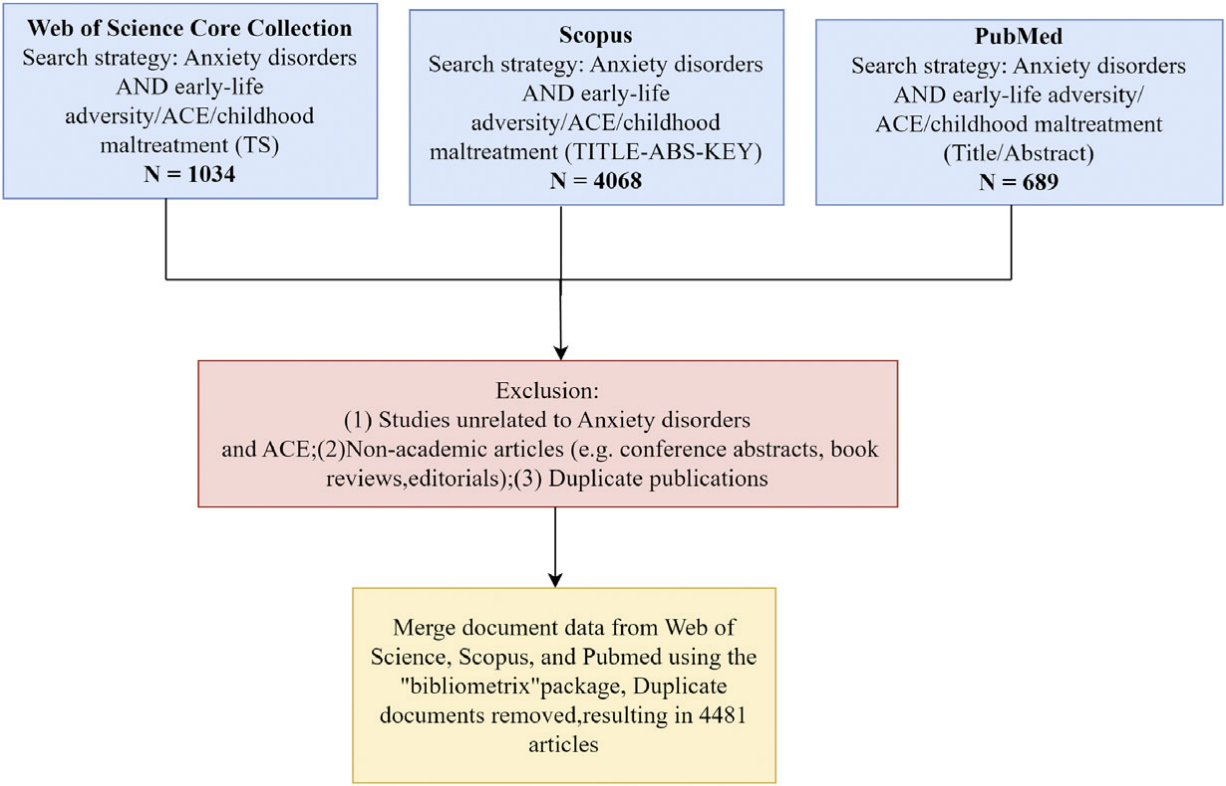
Flow diagram of literature screening and selection. Figure 1. long description.

This study focuses on anxiety disorders and early life adversity (early-life adversity/adverse childhood experiences [ACEs]). We systematically searched three major literature databases: Web of Science Core Collection (WoSCC), Scopus and PubMed. WoSCC includes the Social Sciences Citation Index (SSCI), the Science Citation Index Expanded (SCIE) and the Arts and Humanities Citation Index (A&HCI). Its citation information structure is standardized, with a long time span, making it widely used in bibliometric and citation network analysis research (Yang et al., 2022). Scopus, a comprehensive citation database maintained by Elsevier, is advantageous in terms of journal quantity and international coverage, making it suitable for interdisciplinary research output analysis. PubMed, maintained by the US National Library of Medicine, primarily includes literature in biomedical and life sciences, offering significant reference value in medical and public health research, though its citation information is relatively limited (Falagas et al., 2008; He et al., 2025). The retrieval strategy was constructed around two sets of keywords: one related to anxiety disorders and the other to early life adversity. These two sets of terms were combined using the Boolean operator “AND” to obtain literature addressing both the research subjects and the exposure factors (Chen, 2017; Liu et al., 2024). The full retrieval strings for each database are provided in Supplementary Table S1. The retrieval fields were set as follows: WoSCC used the “Topic” field (TS), Scopus used the “Title, Abstract and Keywords” fields (TITLE-ABS-KEY) and PubMed searched within the “Title and Abstract” fields. All documents were restricted to the English language. For WoSCC and Scopus, document types were limited to Article and Review during the retrieval phase; PubMed did not restrict document types during retrieval but applied this filter in the subsequent data processing phase. The literature screening and integration process is illustrated in Figure 1.

Although Google Scholar, Dimensions and PsycINFO can also be used for topic retrieval, they were not included in the main analysis of this study. The primary reason is that, despite their broad coverage, Google Scholar and Dimensions have limitations in terms of metadata standardization, stability of search results and reproducibility for bibliometric analysis (Harzing and van der Wal, 2008; Thelwall, 2018). PsycINFO, while an important database in the field of psychology, was not included because Web of Science Core Collection (WoSCC) and Scopus already cover a substantial number of psychology and interdisciplinary journals, and PubMed further complements the dataset with biomedical and psychiatric literature. Therefore, this study adopted a combination of WoSCC, Scopus and PubMed to ensure comprehensive topic coverage while controlling methodological complexity and minimizing excessive overlap among databases.

### Data export and merging

After retrieval, literature records were exported from WoSCC, Scopus and PubMed. Specifically, WoSCC was exported in “Full Record and References” format, Scopus in CSV format and PubMed in Citation Manager (.nbib) format. All data were imported into R software (version 4.5.2) and processed using the bibliometrix package for organization and preprocessing. Literature records from different databases were first merged into a comprehensive dataset, and no duplicates were removed at this stage. It is important to note that although WoSCC data includes complete citation information, it was only used for basic bibliographic data integration in the merging phase and was not involved in citation relationship analysis.

### Duplicate detection and document type filtering

After exporting the search results, all records retrieved from WoSCC, Scopus and PubMed were first merged into a unified dataset. A deduplication process was then conducted: the DOI was used as the primary matching field, and for records with missing or inconsistent DOIs, manual verification was performed based on article titles. When necessary, additional information, such as authors, publication year and source journal, was further considered to assist in the identification process, ensuring that only one unique record was retained for each publication.

Following deduplication, document types were uniformly filtered to include only Articles and Reviews, while Editorials, Letters, Comments and other non-research publications were excluded. All of the above procedures were performed during the data preprocessing stage to ensure consistency in the subsequent bibliometric analysis. The detailed workflow is presented in Figure 1.

### Bibliometric analysis

Based on the final cleaned dataset, bibliometric analysis was systematically conducted, including: (1) Annual publication trends in the research field; (2) Distribution of publications by country and region; (3) Publication patterns and collaboration among institutions; (4) Author publication characteristics; and (5) Journal distribution characteristics.

### Keyword co-occurrence and research hotspot analysis

This study used CiteSpace (Chen, 2004), VOSviewer and bibliometrix (Arruda et al., 2022) tools for keyword-related analysis. VOSviewer and bibliometrix performed keyword co-occurrence and institutional collaboration network analysis based on the merged datasets from the three databases; CiteSpace was used exclusively for research hotspot and frontier evolution analysis based on WoSCC data. CiteSpace was primarily used for citation burst analysis and knowledge structure visualization to identify keywords, literature and research themes with significant time-based bursts. The parameters set for CiteSpace included: data source from WoSCC, time span from 2015 to 2025 and network similarity metric using Cosine. VOSviewer focused on constructing and visualizing networks based on co-occurrence relationships, including keyword co-occurrence and institutional collaboration networks. Full counting was used for the counting method, with a minimum frequency of 10 for keywords to enhance the interpretability of the network structure (Chen and Song, 2019).

### Citation relationship analysis

Since citation relationship analysis relies on complete and standardized reference data, and PubMed does not provide systematic citation information, all citation relationship analyses in this study were conducted exclusively using WoSCC data. Specific analyses included co-citation analysis of documents, authors and journals to reveal the knowledge base and academic structure of the research field. It is important to emphasize that citation analysis reflects the structural position and relative influence of documents in the academic network, rather than the quality of the research itself. Given the differences in citation coverage and statistical rules across databases, this study excluded Scopus and PubMed data from citation relationship analysis to avoid potential systematic biases. Overall, this study adopted a layered analysis strategy to ensure a broad coverage of literature while enhancing the stability and methodological consistency of the citation analysis results (Li et al., 2025).

## Results

### Literature screening and data synthesis

As shown in Figure 1, this study systematically searched three major databases for literature related to anxiety disorders and ACE. A total of 5,791 articles were retrieved in the initial search, with 1,034 articles from Web of Science Core Collection (WoSCC), 4,068 from Scopus and 689 from PubMed. After merging the literature records from the three databases and applying predefined inclusion and exclusion criteria, we excluded irrelevant articles, nonacademic literature (such as conference abstracts, book reviews and editorials) and duplicate records. After de-duplication and filtering, a total of 4,481 articles were retained for further bibliometric analysis.

### Annual publication trends in childhood adversity and anxiety research

**Figure 2.**
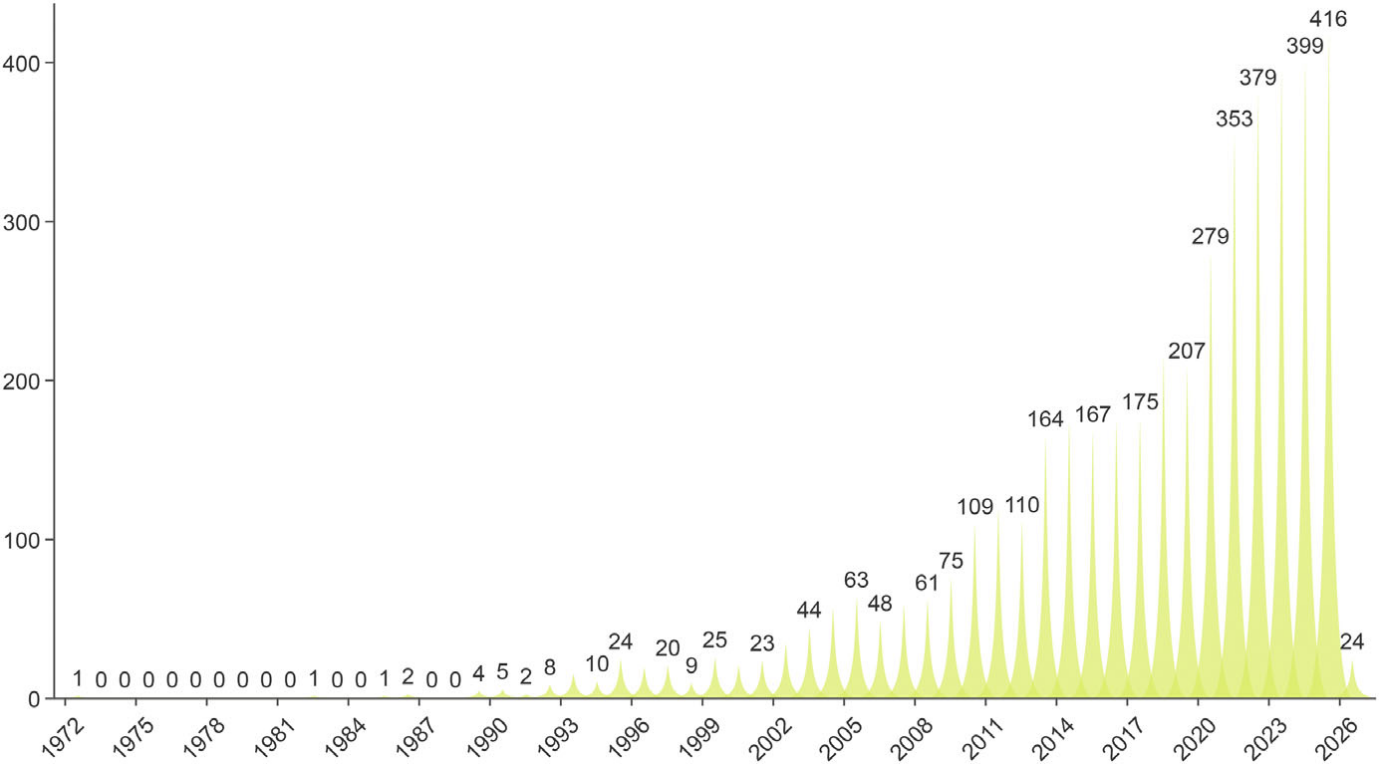
Annual publication trends in the field of childhood adversity and anxiety research (1972–2026). Figure 2. long description.

As shown in Figure 2, the annual publication volume on anxiety disorders and early life adversity demonstrates clear phase-based growth. From the 1970s to the early 1980s, research in this field was in its early stages, with very few publications and sporadic outputs in most years. Starting in the 1990s, relevant studies began to emerge, with a slow increase in annual publications, although the overall scale remained limited. After 2000, the publication volume steadily increased, with a significant acceleration after 2010, indicating that this research topic has gradually gained widespread attention in the academic community. Over the past decade, annual publication volumes have significantly surged and have reached their peak in recent years, reflecting that anxiety disorders and early life adversity have become important research hotspots in mental health and public health. Overall, the field has experienced rapid growth following long-term accumulation.

### Distribution of publications and collaboration patterns by country

Table 1 shows the countries with the highest publication volumes in research on anxiety disorders and early life adversity, along with their international collaboration characteristics. The United States dominates the field with 1,385 publications, accounting for 30.9% of the total output, significantly higher than other countries. Of these, 1,327 articles were single-country collaborations (SCPs), and 58 were multicountry collaborations (MCPs), making up 4.2% of the total. Canada (294 articles, 6.6%) and China (251 articles, 5.6%) follow behind. Notably, China’s MCP proportion (11.2%) is higher than that of Canada (7.8%), indicating higher international collaboration participation. The United Kingdom (226 articles, 5.0%) and Germany (221 articles, 4.9%) have similar publication volumes, with Germany’s MCP proportion (12.2%) slightly higher than that of the United Kingdom (7.1%). Other high-output countries, such as the Netherlands (188 articles, 4.2%) and Australia (180 articles, 4.0%), have MCP proportions near or exceeding 8%. Although France (12.7%) and Brazil (20.5%) have relatively low total publication volumes, their high MCP proportions suggest strong international collaboration in this research area.

**Table 1.** National contributions and international collaboration patterns in research on childhood adversity and anxiety Table 1. long description.

Country	Articles	Articles %	SCP	MCP	MCP %
USA	1,385	30.9	1,327	58	4.2
CANADA	294	6.6	271	23	7.8
CHINA	251	5.6	223	28	11.2
UNITED KINGDOM	226	5	210	16	7.1
GERMANY	221	4.9	194	27	12.2
NETHERLANDS	188	4.2	173	15	8
AUSTRALIA	180	4	163	17	9.4
ITALY	108	2.4	101	7	6.5
FRANCE	79	1.8	69	10	12.7
BRAZIL	78	1.7	62	16	20.5

### Top 10 journals, authors and institutions in childhood adversity and anxiety research

**Figure 3.**
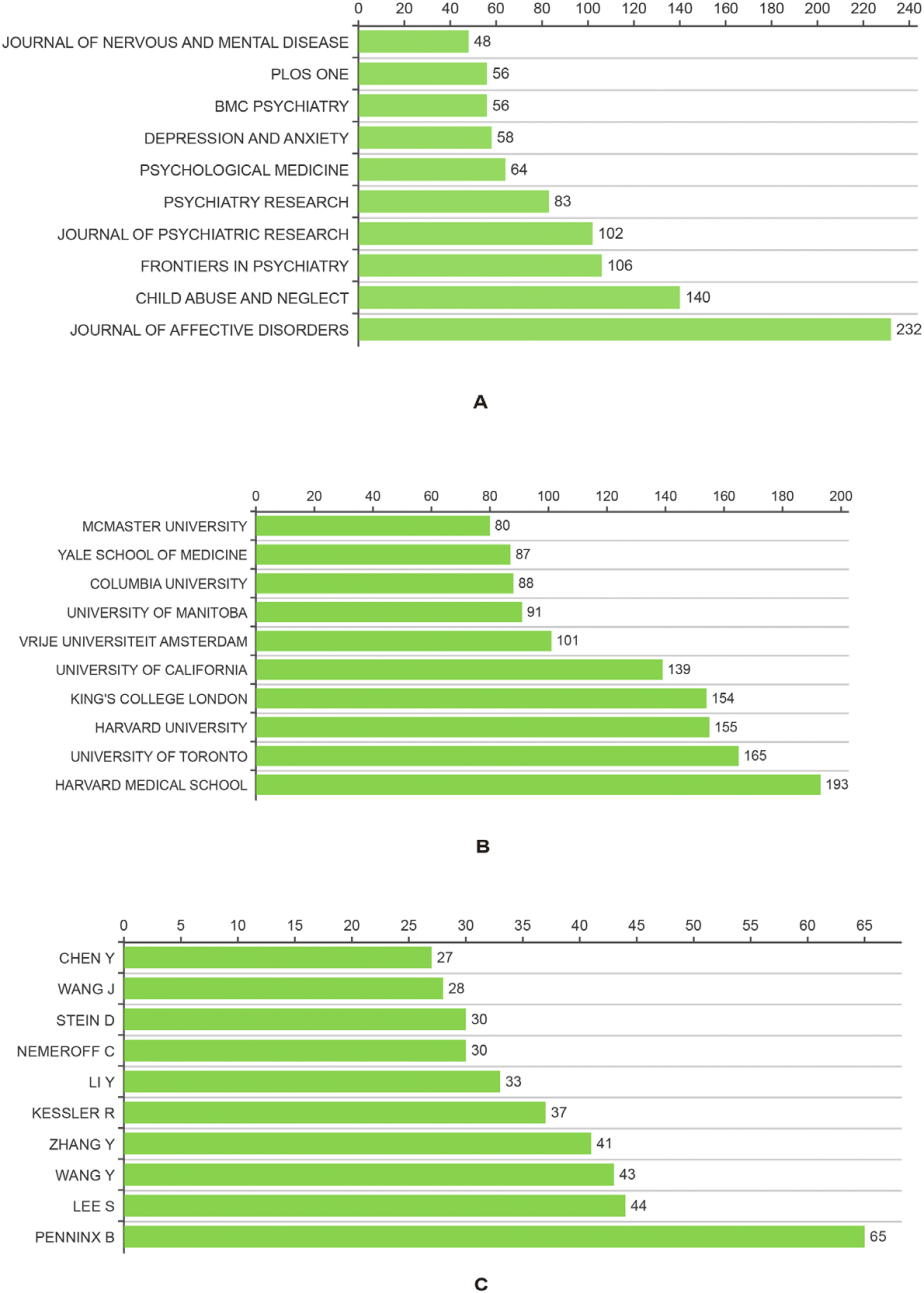
Top 10 journals, institutions and authors in childhood adversity and anxiety research across three databases. (A) Top 10 journals by publication count in childhood adversity and anxiety research. (B) Top 10 institutions by publication count in childhood adversity and anxiety research. (C) Top 10 authors by publication count in childhood adversity and anxiety research. Rankings are based on the total number of publications retrieved from the three databases. Figure 3. long description.

As shown in Figures 3A–3C, this study conducted statistical analyses of the high-output journals, institutions and authors in research on anxiety disorders and early life adversity. Figure 3A (Journal distribution) shows that research results are primarily published in journals related to psychiatry and mental health. The *Journal of Affective Disorders* has the highest publication volume (232 articles), significantly ahead of other journals, followed by *Child Abuse & Neglect* (140 articles) and *Frontiers in Psychiatry* (106 articles). Other journals, such as the *Journal of Psychiatric Research*, *Psychiatry Research*, *Psychological Medicine* and *Depression and Anxiety*, also published a considerable number of related studies, indicating that research in this field is mainly concentrated in psychiatry, psychology and related interdisciplinary journals. Figure 3B (Institution distribution) shows that high-output institutions are mainly concentrated in North America and Europe. Harvard Medical School (193 articles) and the University of Toronto (165 articles) rank at the top, followed by Harvard University (155 articles) and King’s College London (154 articles). In addition, the University of California system, Vrije Universiteit Amsterdam and Columbia University also show high research output in this field. Figure 3C (Author distribution) presents high-output authors. Penninx B ranks first with 65 publications, significantly ahead of other authors. Following are Lee S (44 articles), Wang Y (43 articles) and Zhang Y (41 articles). Other high-output authors such as Kessler R, Li Y and Nemeroff C have similar publication volumes, suggesting that the field is led by a few core authors with multiple research teams contributing to the studies.

### Keyword analysis of childhood adversity and anxiety research based on the bibliometrix package

**Figure 4.**
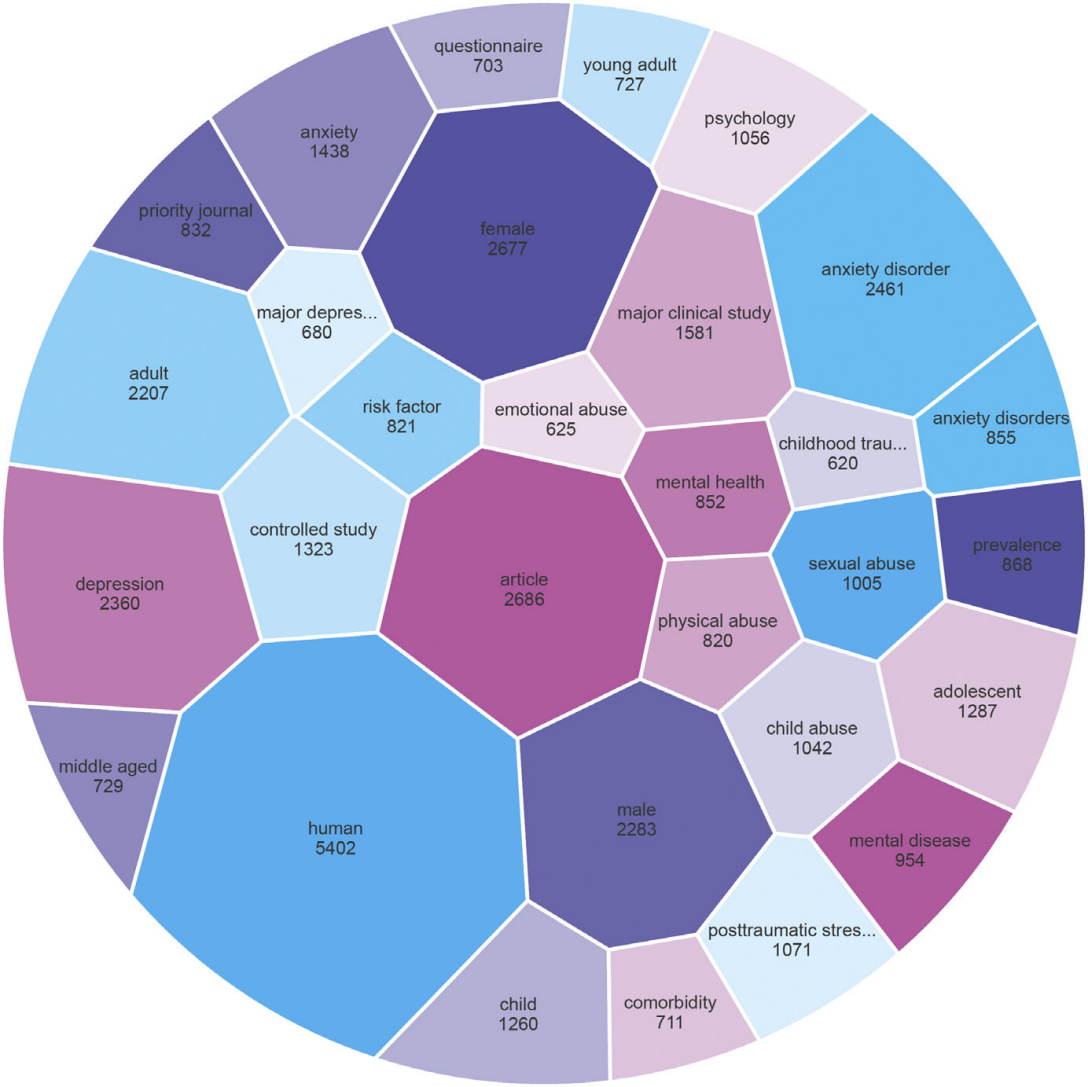
Top 30 high-frequency keywords in studies on childhood adversity and anxiety. Figure 4. long description.

As shown in Figure 4, the figure displays the top 30 most frequent keywords in the study, with their corresponding frequencies. It does not involve keyword clustering or thematic classification. Overall, high-frequency keywords mainly focus on the research subjects, population characteristics, mental disorder terminology and terms related to childhood adversity. Among the research subjects and population-related terms, keywords such as “human,” “female,” “male,” “adult,” “child” and “adolescent” appear frequently, reflecting that the research in this field is primarily based on human samples covering different genders and age groups. In terms of mental disorder terminology, keywords like “anxiety disorder(s),” “depression,” “major depression,” “mental health” and “mental disease” are frequently seen, indicating that anxiety disorders and related emotional disorders are the most discussed research subjects. For childhood adversity-related terms, keywords such as “child abuse,” “emotional abuse,” “physical abuse,” “sexual abuse” and “childhood trauma” appear frequently, suggesting that various forms of childhood adversity are widely studied. Additionally, keywords like “posttraumatic stress,” “comorbidity,” “risk factor” and “prevalence” reflect that research often involves the epidemiological characteristics and associated risk factors. Overall, the figure provides an intuitive representation of the most frequently used keywords and their frequency distribution in the research field.

### Institutional collaboration and keyword co-occurrence network analysis based on VOSviewer

**Figure 5.**
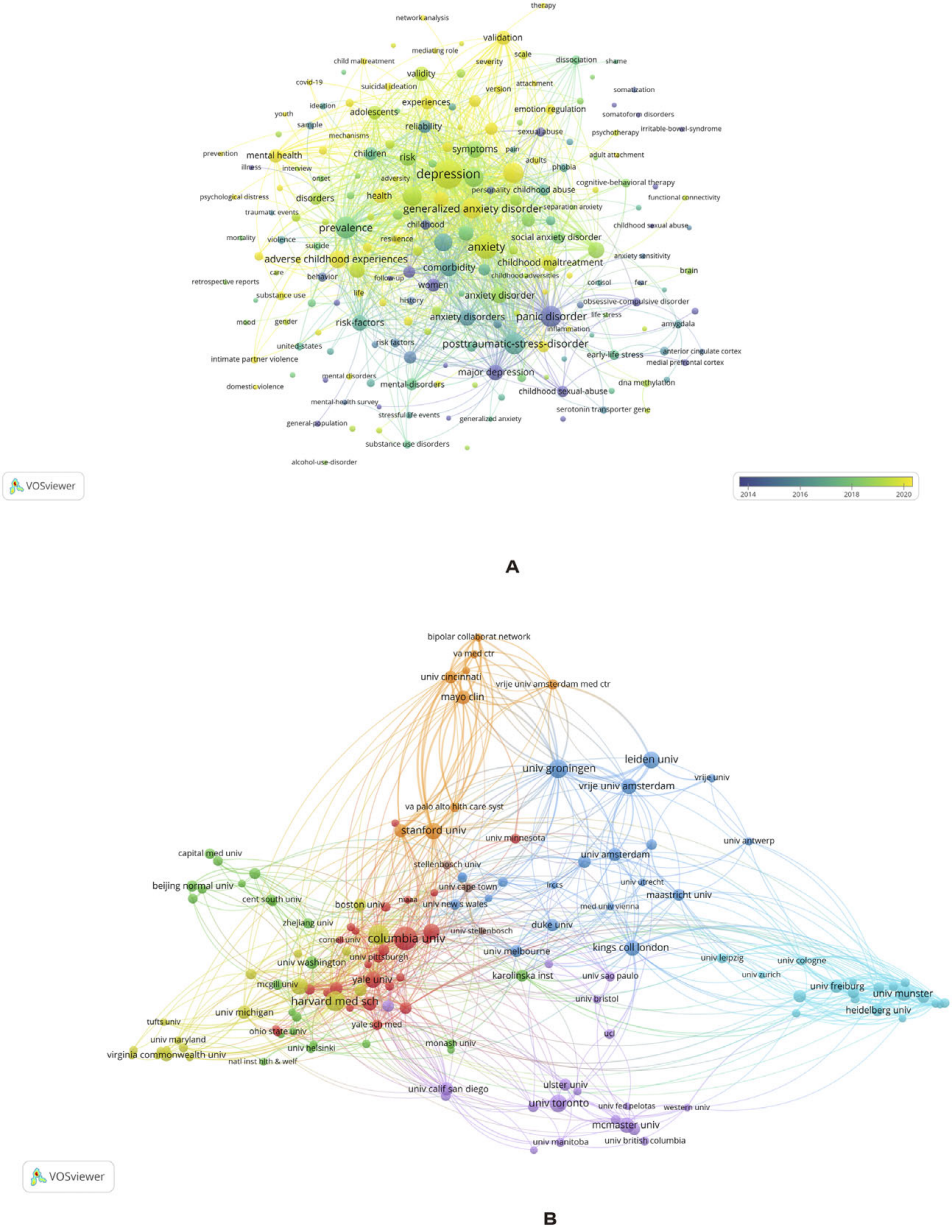
Network visualization of childhood adversity and anxiety research. (A) Keyword co-occurrence network highlighting core themes related to childhood adversity and anxiety, including anxiety disorders, depression and trauma-related outcomes. (B) Institutional collaboration network depicting co-authorship patterns in the field of childhood adversity and anxiety research. Figure 5. long description.

Figure 5 shows the keyword co-occurrence and time overlay, as well as institutional collaboration network visualization. Figure 5A presents the keyword co-occurrence network and time overlay visualization results based on VOSviewer. In the figure, nodes represent keywords, with node size proportional to keyword frequency. The lines between nodes represent the strength of keyword co-occurrence within the same article, and the color reflects the average publication year, transitioning from blue (earlier) to yellow (later). The overall network structure shows a highly clustered configuration centered around “depression” and “anxiety,” closely connected with keywords like “adverse childhood experiences,” “childhood maltreatment,” “posttraumatic stress disorder” and “generalized anxiety disorder,” forming the core research themes. The time overlay results show that early research (blue) primarily focused on specific clinical phenotypes of PTSD, panic disorder and major depression. In recent years (yellow), the research hotspots have shifted toward topics such as adverse childhood experiences, emotion regulation, resilience, validation and psychosocial mechanisms, indicating a shift from single disease diagnoses to integrated research on early adversity exposure, cross-diagnostic risk factors and underlying psychological mechanisms.

Figure 5B shows the institutional collaboration network in this research area. Nodes represent research institutions, with node size reflecting publication volume and lines indicating collaboration between institutions. Different colors represent different collaboration clusters. The network structure reveals clear multicenter and cross-regional characteristics. Clusters centered around institutions such as Columbia University, Harvard Medical School and Stanford University hold significant positions in the network, indicating high research output and collaboration intensity. European institutions, such as Vrije Universiteit Amsterdam, Leiden University and the University of Groningen, form relatively tight subnetworks and maintain frequent cross-national collaboration with North American institutions. Additionally, universities in Asia and Oceania are gradually integrating into the international collaboration network, but their overall connectivity remains lower. This result suggests that research in this field has formed an international collaboration pattern dominated by high-level research institutions in Europe and North America, but there is still room to further deepen global cooperation.

### Keyword clustering and citation burst analysis based on CiteSpace

**Figure 6.**
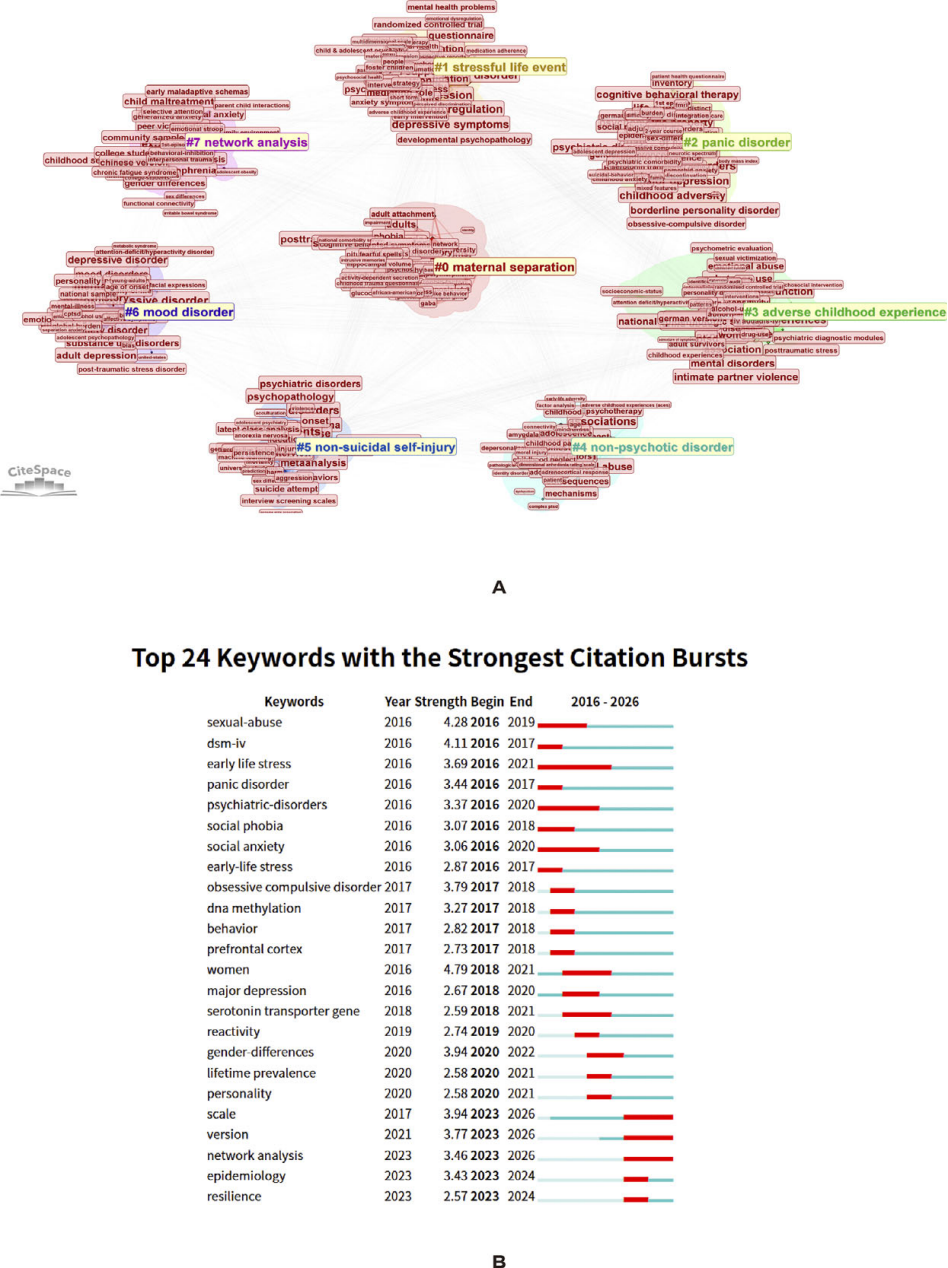
Keyword clustering and citation burst analysis of childhood adversity and anxiety research based on the Web of Science Core Collection. (A) Keyword clustering network generated by CiteSpace, illustrating major research themes in childhood adversity and anxiety, with clusters labeled according to dominant terms. (B) Top 24 keywords with the strongest citation bursts from 2016 to 2026, indicating emerging and evolving research frontiers in childhood adversity- and anxiety-related studies. Analyses were conducted using publications retrieved from the Web of Science Core Collection. Figure 6. long description.

Figure 6A displays the keyword co-occurrence clustering network generated by CiteSpace. Nodes in the network represent keywords, with node size related to keyword frequency, and lines represent the co-occurrence relationship between keywords. Different-colored convex hulls represent different thematic clusters, with labels based on the most representative keywords within the clusters. Several clearly structured and focused keyword clusters were identified, including: #0 “maternal separation,” which focuses on mother–infant separation, attachment patterns and early caregiving deprivation, reflecting the long-term impact of early parent–child relationship disruption on subsequent mental health; #1 “stressful life event,” focusing on stressful life events, emotion regulation and depressive symptoms, emphasizing the role of environmental stress in the development of mental disorders; #2 “panic disorder,” involving panic disorder, cognitive behavioral therapy and comorbidity issues, representing a more established clinical research direction; #3 “adverse childhood experience,” focusing on childhood adversity, domestic violence, intimate partner violence and post-traumatic stress response, forming the core of current research; #4 “nonpsychotic disorder,” covering nonpsychotic disorders, symptom mechanisms and psychological treatment-related themes; #5 “nonsuicidal self-injury,” focusing on nonsuicidal self-injury, adolescent psychopathology and risk assessment; #6 “mood disorder,” including depressive disorders, bipolar disorder and emotional regulation abnormalities; #7 “network analysis,” reflecting the recent methodological evolution in research, emphasizing symptom networks and the introduction of complex systems perspectives. Overall, the keyword clustering presents a clear structure, evolving from early environmental exposures (such as maternal separation and childhood adversity) to specific mental disorder phenotypes (anxiety, depression and panic disorder), and later expanding to methodological and mechanistic research (network analysis), reflecting the multilayered and interdisciplinary nature of the research themes.

Figure 6B displays the 24 keywords with the highest citation burst intensity between 2016 and 2026. Red bars indicate keywords that saw a significant rise in frequency during the corresponding time period, with burst intensity (Strength) reflecting the degree of research attention. The results show that early burst keywords primarily include “sexual abuse,” “DSM-IV,” “early life stress,” “panic disorder” and “psychiatric disorders,” indicating that early research focused on defining childhood trauma types and diagnostic classifications of mental disorders. Later, the research focus shifted toward biological mechanisms, including “DNA methylation,” “prefrontal cortex” and “serotonin transporter gene,” reflecting the rise of molecular genetics and neurobiology in this field. Notably, recent and ongoing burst keywords include “network analysis,” “epidemiology,” “resilience,” “scale” and “version,” indicating that current research is transitioning from single risk factors or disease diagnoses to complex systems modeling, population-level epidemiological features and optimizing psychological resilience and measurement tools. CiteSpace analysis shows that the research theme in this field has evolved from early trauma types and diagnostic classifications to biological mechanism exploration, and in recent years, it has clearly shifted toward integrated research directions involving network analysis, epidemiology and resilience.

### Citation and local citation analysis of the top 10 countries, journals and authors in childhood adversity and anxiety research

**Figure 7.**
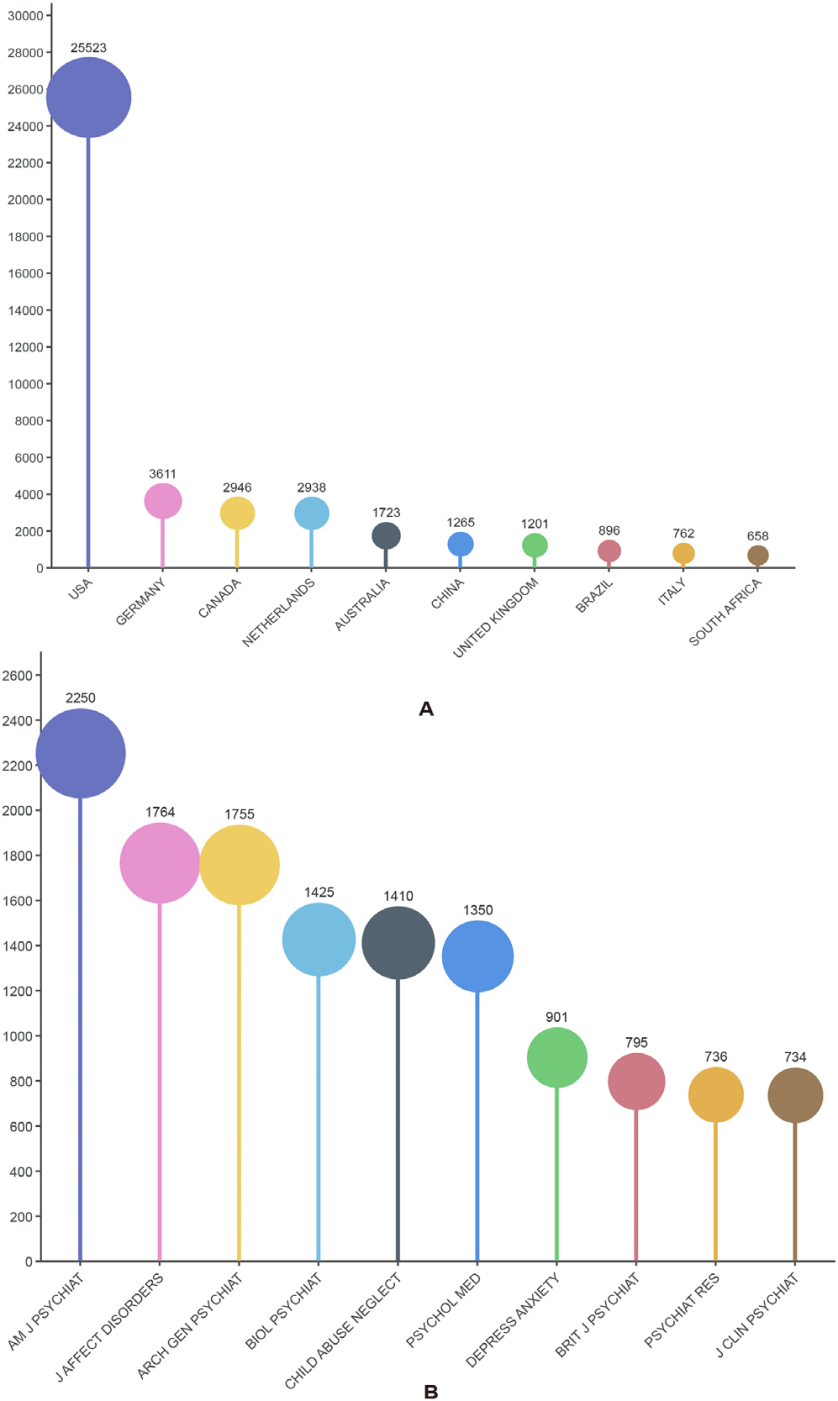
Citation analysis of childhood adversity and anxiety research based on the Web of Science Core Collection. (A) Top 10 countries by total citations in childhood adversity and anxiety research. (B) Top 10 journals by total citations in childhood adversity and anxiety research. Data were retrieved from the Web of Science Core Collection, and citation counts reflect cumulative citations within the study period. Figure 7. long description.

Figure 7A shows the distribution of citation frequencies by publishing country. The United States holds an absolute dominance in this field, with the highest total citations (25,523), significantly ahead of other countries, reflecting its central academic influence in the field. Germany (3,611 citations), Canada (2,946 citations), the Netherlands (2,938 citations) and Australia (1,723 citations) form the second tier. China (1,265 citations) and the United Kingdom (1,201 citations) follow closely, while Brazil, Italy and South Africa have relatively low citation frequencies. Overall, the citation distribution shows clear imbalances, suggesting that high-impact research in this field is mainly concentrated in a few developed countries.

Figure 7B shows the journals with the highest citation frequencies in the field. The *American Journal of Psychiatry* has the highest number of citations (2,250), playing a significant academic leadership role in the field. Following are the *Journal of Affective Disorders* (1,764 citations) and *Archives of General Psychiatry* (1,755 citations), reflecting the central position of journals in emotional disorders and psychiatry in this field. Additionally, journals such as *Biological Psychiatry*, *Child Abuse & Neglect* and *Psychological Medicine* have high citation frequencies, indicating the high intersection of research in clinical psychiatry, biological psychiatry and childhood adversity. Overall, high-citation journals are mainly authoritative journals in psychiatry and psychology, indicating that the research topic holds a strong influence in mainstream psychiatric fields.

**Figure 8.**
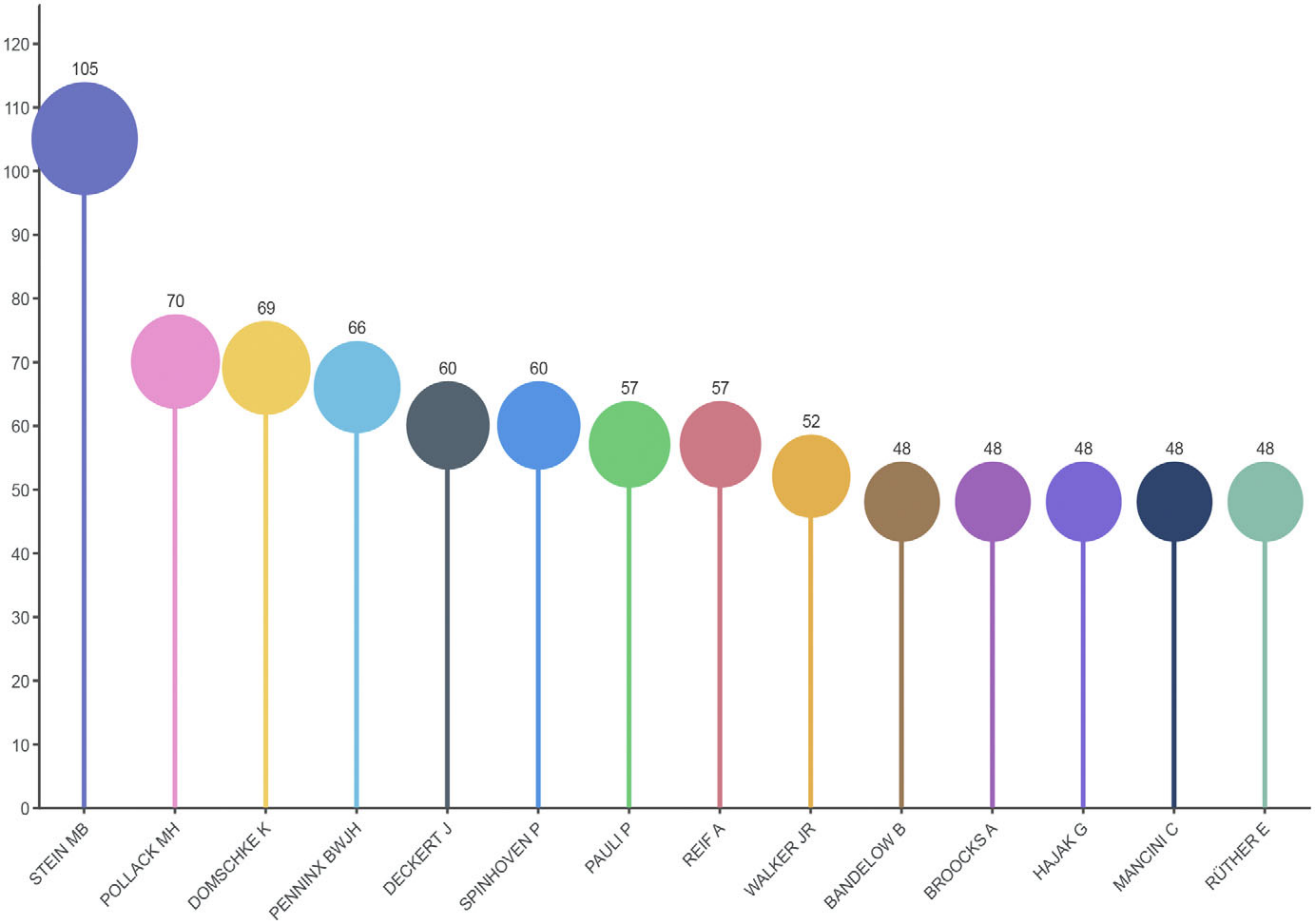
Top 10 most cited authors in childhood adversity and anxiety research based on the Web of Science Core Collection. The figure shows the top 10 authors ranked by total citation counts in childhood adversity and anxiety research. Citation data were retrieved from the Web of Science Core Collection, and counts represent cumulative citations within the study period. Figure 8. long description.

Figure 8 displays the authors with the highest citation frequencies. Stein MB has the highest citation frequency (105 citations), ranking first, showing his sustained academic impact in the field. Following him are Pollack MH (70 citations), Domschke K (69 citations), Pine DS (66 citations) and Deckert J (60 citations). These scholars have long been involved in researching anxiety disorders, emotional disorders and related biological and psychological mechanisms. Overall, high-citation authors are concentrated in psychiatry and clinical psychology, with their research themes highly correlated with anxiety, depression and early life adversity, further confirming the stability and continuity of the core research community in this field.

### International and local citation analysis of childhood adversity and anxiety research

Based on global citation statistics, Table 2 lists the top 10 most cited representative articles in this field. The high-citation articles are mainly focused on the following core areas: (1) Neurobiological mechanisms of anxiety and trauma-related disorders: The most cited article is a functional neuroimaging meta-analysis by Etkin and Wager (2007) published in the *American Journal of Psychiatry* (cited 2,521 times), which integrates brain function abnormalities in PTSD, social anxiety disorder and specific phobias in emotional processing, laying the foundational framework for neuroimaging research on anxiety disorders (Etkin and Wager, 2007). Other articles, such as those by Bremner et al. (1999), Milad et al. (2009) and Shin and Liberzon (2010), further deepen the understanding of the amygdala–prefrontal cortex–hippocampus pathway in PTSD and anxiety disorders, constituting classic references in neurobiological research in this field (Milad et al., 2009; Shin and Liberzon, 2010). (2) Childhood adversity and mental disorder risk: The systematic review and meta-analysis by Chen et al. (2010) (cited 730 times) clearly points out the significant association between childhood sexual abuse and the lifetime risk of multiple mental disorders, serving as a key evidence base in childhood adversity research (Chen et al., 2010). Additionally, McLaughlin et al. (2010) “stress sensitization hypothesis” provided a theoretical foundation for numerous subsequent mechanistic and longitudinal studies (McLaughlin et al., 2010). (3) Comorbidity of anxiety and depression and the cross-diagnostic perspective: Lamers et al. (2011) revealed a high comorbidity pattern between anxiety and depression in the NESDA cohort (cited 693 times), emphasizing the limitations of the traditional single-diagnosis framework (Lamers et al., 2011). This trend was further developed in the “p factor” model proposed by Caspi and Moffitt (2018) (cited 665 times), representing a significant milestone in the transition from categorical diagnosis to cross-diagnostic dimensional models (Caspi and Moffitt, 2018). (4) Personality disorders and comorbidity structures: Early high-citation articles, such as Zanarini et al. (1998) on Axis I comorbidity patterns in borderline personality disorder (cited 673 times), reflect the ongoing focus on complex psychopathological structures in the field (Zanarini et al., 1998).

**Table 2.** Top 10 globally most cited publications in the field of childhood adversity and anxiety Table 2. long description.

Number	First author	Article name	Source	Year	Global citations
1	Amit Etkin	Functional neuroimaging of anxiety: a meta-analysis of emotional processing in PTSD, social anxiety disorder, and specific phobia	Am J Psychiatry	2007	2,521
2	Lisa M Shin	The neurocircuitry of fear, stress, and anxiety disorders	Neuropsychopharmacology	2010	1,486
3	Mohammed R Milad	Neurobiological basis of failure to recall extinction memory in posttraumatic stress disorder	Biol Psychiatry	2009	1,039
4	Laura P Chen	Sexual abuse and lifetime diagnosis of psychiatric disorders: systematic review and meta-analysis	Mayo Clin Proc	2010	730
5	Femke Lamers	Comorbidity patterns of anxiety and depressive disorders in a large cohort study: the Netherlands Study of Depression and Anxiety (NESDA)	J Clin Psychiatry	2011	693
6	M C Zanarini	Axis I comorbidity of borderline personality disorder	Am J Psychiatry	1998	673
7	Avshalom Caspi	All for One and One for All: Mental Disorders in One Dimension	Am J Psychiatry	2018	665
8	K A McLaughlin	Childhood adversity, adult stressful life events, and risk of past-year psychiatric disorder: a test of the stress sensitization hypothesis in a population-based sample of adults	Psychol Med	2010	643
9	J D Bremner	Neural correlates of exposure to traumatic pictures and sound in Vietnam combat veterans with and without posttraumatic stress disorder: a positron emission tomography study	Biol Psychiatry	1999	513
10	Charles B Nemeroff	Posttraumatic stress disorder: a state-of-the-science review	J Psychiatr Res	2006	478

Table 3 lists the top 10 most cited articles in the local literature sample of this study. Compared with global high-citation articles, local high-citation articles have a more focused and consistent thematic structure, mainly concentrating on the link between childhood adversity/abuse and anxiety disorders. (1) Clinical association between childhood abuse and anxiety disorders: The leading articles (Safren SA, Etkin A and Simon NM, each cited 35 times) systematically explore the relationship between childhood abuse history and panic disorder, social anxiety disorder and generalized anxiety disorder, emphasizing the close connection between adverse childhood experiences and the severity of anxiety symptoms, functional impairment and decreased quality of life (Safren et al., 2002; Etkin and Wager, 2007; Simon et al., 2009). These studies are mostly based on clinical samples or large epidemiological surveys, providing empirical evidence for childhood adversity as a significant risk factor for anxiety disorders. (2) Anxiety disorder subtypes and adversity-specific effects: Two studies by Bandelow et al. (2002, 2004) are ranked in the top 10 in local citations, analyzing the differential roles of various early traumas, parental rearing styles and family psychiatric history in social anxiety disorder and panic disorder, suggesting that childhood adversity may not have a homogeneous effect on all anxiety disorders, but could have disorder-specific pathways (Bandelow et al., 2002, 2004). (3) Population-level evidence and mechanism hypotheses: Studies by Goodwin et al. (2005) and Cougle et al. (2010) based on National Comorbidity Survey data further confirm the independent association between childhood physical and sexual abuse and the risk of panic attacks and anxiety disorders in adulthood (Bandelow et al., 2002; Goodwin et al., 2005). The stress sensitization hypothesis proposed by McLaughlin (2010) is frequently cited in the local literature, reflecting its theoretical integration in explaining childhood adversity and subsequent mental disorder susceptibility (McLaughlin et al., 2010). (4) Early manifestation of the cross-diagnostic perspective: Spinhoven P’s (2010) study emphasized the shared and specific effects of childhood adversity and negative life events in anxiety and depression disorders, providing early empirical support for subsequent cross-diagnostic and dimensional research (Spinhoven et al., 2010).

**Table 3.** Top 10 most cited publications in the field of childhood adversity and anxiety Table 3. long description.

Number	First author	Article name	Source	Year	Local citations
1	Steven A Safren	History of childhood abuse in panic disorder, social phobia, and generalized anxiety disorder	J Nerv Ment Dis	2002	35
2	Amit Etkin	Functional neuroimaging of anxiety: a meta-analysis of emotional processing in PTSD, social anxiety disorder, and specific phobia	Am J Psychiatry	2007	35
3	Naomi M Simon	Childhood maltreatment linked to greater symptom severity and poorer quality of life and function in social anxiety disorder	Depress Anxiety	2009	35
4	Borwin Bandelow	Early traumatic life events, parental rearing styles, family history of mental disorders, and birth risk factors in patients with social anxiety disorder	Eur Arch Psychiatry Clin Neurosci	2004	27
5	Jesse R Cougle	Examining the unique relationships between anxiety disorders and childhood physical and sexual abuse in the National Comorbidity Survey-Replication	Psychiatry Res	2010	27
6	Renee D Goodwin	Childhood abuse and familial violence and the risk of panic attacks and panic disorder in young adulthood	Psychol Med	2005	26
7	Laura P Chen	Sexual abuse and lifetime diagnosis of psychiatric disorders: systematic review and meta-analysis	Mayo Clin Proc	2010	24
8	K A McLaughlin	Childhood adversity, adult stressful life events, and risk of past-year psychiatric disorder: a test of the stress sensitization hypothesis in a population-based sample of adults	Psychol Med	2010	24
9	Philip Spinhoven	The specificity of childhood adversities and negative life events across the life span to anxiety and depressive disorders	J Affect Disord	2010	23
10	Borwin Bandelow	Early traumatic life events, parental attitudes, family history, and birth risk factors in patients with panic disorder	Compr Psychiatry	2002	21

## Discussion

### Overall development trend of childhood adversity and anxiety research

This study systematically conducted a bibliometric analysis of research on childhood adversity and anxiety disorders based on three major international databases. The results show that this field has undergone an evolution from long-term slow accumulation to rapid growth in the past two decades. Since 2000, the number of related studies has consistently increased, with a notable acceleration after 2010, reflecting the growing academic recognition of childhood adversity as a significant risk factor for anxiety disorders. This development trend aligns closely with the shift in the paradigm of mental health research, where the focus has gradually shifted from adult symptomatology to emphasizing the long-term impact of early life experiences on the onset and development of mental disorders.

It is worth noting that the rapid growth in publication volume has not been driven by a single discipline but is the result of the interdisciplinary integration of psychiatry, psychology, public health and neuroscience, providing a more multidimensional research perspective for understanding the complex etiology of anxiety disorders. At the same time, international attention to mental health issues has steadily increased in the past two decades, especially after the COVID-19 pandemic, with global multicenter cohort studies, longitudinal follow-up studies and big data analysis rapidly accumulating (Madigan et al., 2023). Existing systematic reviews and empirical studies indicate that anxiety levels among children and adolescents generally increased during the pandemic, and individuals with a history of childhood adversity showed higher anxiety risk when faced with pandemic-related stress (Lian et al., 2024; Kalia and Knauft, 2025). Furthermore, the standardization of ACE assessment tools, the deepening of multidisciplinary collaboration models and the continued emphasis on adolescent mental health in public health policy have all contributed to the rapid development of this research area (Singirankabo et al., 2025).

### Country and institution distribution: Research landscape dominated by Europe and North America

Analysis of the country and institution distribution shows that the United States holds a dominant position in childhood adversity and anxiety research, not only leading in publication volume and citation frequency but also occupying central positions in high-impact journals and core research institutions. Notably, the research framework represented by ACE was first proposed and promoted in the United States, laying a crucial foundation for subsequent large-scale population studies and interdisciplinary expansion (Felitti et al., 1998; Anda et al., 2006). Additionally, sustained funding from institutions such as the National Institutes of Health (NIH) for mental health and life course research has significantly facilitated high-output and high-impact studies in this field (Insel, 2009).

At the same time, European countries such as Germany, the Netherlands and the United Kingdom have shown outstanding performance in terms of publication impact and international collaboration, forming a relatively stable and high-quality research network. This may be related to their emphasis on multicenter collaboration, cross-national cohort sharing and public health-oriented research models (Viner et al., 2012). In contrast, while developing countries such as China have experienced rapid growth in publication volume in recent years, their overall citation frequency and international influence still need improvement, suggesting that there remains a regional imbalance in childhood adversity and anxiety research globally. This also reflects the uneven distribution of research resources, data foundations and international collaboration opportunities. This geographic distribution pattern suggests that the existing body of evidence may predominantly reflect patterns of adversity exposure and anxiety phenotypes within Western sociocultural contexts, and therefore, the cross-cultural generalizability of these findings requires further validation. Meanwhile, these results should be interpreted with caution, as factors such as differences in database coverage, language bias and publication practices may also, to some extent, influence the observed geographic disparities.

### Evolution of research themes: From trauma types to cross-diagnosis and mechanistic integration

Based on keyword co-occurrence, clustering and time-overlay analysis results, it is evident that the themes in childhood adversity and anxiety research have shown clear stages of evolution. The research framework in this stage was mainly based on traditional psychiatric classification systems, aiming to identify the risk roles of different trauma experiences in the formation of specific anxiety disorders. Among these, Brewin et al. conducted a meta-analysis systematically summarizing various independent risk factors for post-traumatic stress disorder (PTSD), revealing the stable predictive roles of childhood adversity, previous trauma history and psychiatric history across different populations. They also pointed out that most risk factors had relatively limited effect sizes, while factors during or after trauma (such as trauma severity, lack of social support and additional life stress) had more significant impacts. This study provided an important methodological foundation for early research on the risk factors in childhood adversity and anxiety disorders (Brewin et al., 2000). On this basis, Cougle et al. further explored the specific associations between childhood physical and sexual abuse and different anxiety disorders through data from the National Comorbidity Survey. They found that, after controlling for depression, other anxiety disorders, parental anxiety history and demographic variables, childhood sexual abuse had independent associations with social anxiety disorder, panic disorder, generalized anxiety disorder and PTSD, while the association of physical abuse was more limited, mainly concentrated in PTSD and specific phobias. Moreover, the study also revealed gender differences, suggesting that different types of childhood abuse may affect anxiety outcomes through different pathways in males and females. Overall, the research in this period aimed to clarify “which types of trauma corresponded to which anxiety disorder.” This work, to some extent, contributed to the empirical accumulation of the relationship between childhood adversity and anxiety, and also gradually revealed the limitations of excessive reliance on trauma types and diagnostic categories, paving the way for the subsequent shift toward a comprehensive adversity concept and a cross-diagnostic perspective (Cougle et al., 2010).

As research continued to accumulate, keyword clustering and co-occurrence structures showed that different types of childhood adversity exhibit high co-occurrence and overlap at the psychopathological level, and single trauma or single diagnosis could no longer fully explain the complex presentation of anxiety-related symptoms. In this context, the research focus gradually shifted toward the comprehensive concept of ACE, emphasizing the cumulative effects of childhood adversity and overall risk exposure. This shift was initiated by the pioneering research of Felitti et al., who first systematically proposed the ACE framework. They revealed that multiple forms of childhood abuse and family dysfunction often coexist and show stable dose–response relationships with various health risk behaviors and diseases in adulthood, thus breaking the traditional research paradigm of explaining long-term health outcomes with a single traumatic event (Felitti et al., 1998). Subsequently, Anda et al., based on ACE research, further integrated epidemiological and neurobiological evidence, pointing out that cumulative exposure to ACEs might affect brain structure and function by continuously activating the stress response system, and is broadly associated with emotional, substance use and multisystem health outcomes. This evidence strengthened the theoretical foundation for viewing childhood adversity as a cross-diagnostic and cross-system risk factor (Anda et al., 2006). From a developmental psychology and methodological perspective, the cumulative risk model proposed by Evans et al. provided an important supplement to ACE research, emphasizing that exposure to multiple risk factors has a greater impact on individual development than a single risk factor. This perspective aligns with the bioecological model, the chronic stress homeostasis model and evolutionary developmental theories, providing a more integrative theoretical framework for understanding the widespread and enduring effects of childhood adversity (Evans et al., 2013). Overall, the shift from single trauma types to ACE and cumulative risk models significantly promoted comparative and integrative research across disorders and symptoms, and laid the theoretical foundation for subsequent attention to emotion regulation, psychological resilience and multi-pathway mechanisms.

In recent years, keyword time-overlay and burst analysis further show that the focus of childhood adversity and anxiety research has gradually expanded from traditional diagnostic classifications and specific trauma type descriptions to emotional regulation processes, psychological resilience, cross-diagnostic risk factors and symptom-level analysis frameworks. This change reflects the researchers’ growing focus on the psychological mechanisms between early adversity and anxiety outcomes, rather than just the direct associations between exposure and diagnosis. In this research orientation, emotional susceptibility traits have gradually become a focus of study. McLaughlin et al.’s research, for example, proposed that anxiety sensitivity is an important risk factor for the development of anxiety symptoms in adolescents and adults, and further pointed out that stressful life events could promote the occurrence and exacerbation of anxiety symptoms by increasing anxiety sensitivity. Longitudinal studies have shown that anxiety sensitivity mediates the relationship between stress exposure and changes in anxiety symptoms, and this effect is somewhat specific to anxiety symptoms, not fully applicable to depression symptoms. Such studies provide key psychological evidence for understanding how childhood adversity affects anxiety risk through emotional processing and cognitive response patterns (McLaughlin and Hatzenbuehler, 2009). Meanwhile, research hotspots have gradually extended to the effects of childhood adversity on brain structure and function. McLaughlin et al.’s “deprivation–threat” theoretical framework, proposed in 2014, emphasizes that different types of adverse environmental experiences may affect emotional and behavioral outcomes through different neurodevelopmental pathways (McLaughlin et al., 2014). This framework distinguishes between deprivation (lack of expected environmental input) and threat (involving harm to the individual’s physical or psychological integrity), and points out that using traditional stress response pathways to explain the neural effects of childhood adversity has limitations. This model provides a more integrative theoretical perspective for explaining how childhood adversity affects anxiety-related symptoms through multiple neural and psychological mechanisms. Overall, the shift from emotional regulation traits to neurodevelopmental dimensions reflects the paradigm shift from single diagnosis-oriented research to cross-diagnostic and cross-level mechanism integration, providing a more systematic explanatory framework for understanding the onset, maintenance and heterogeneity of anxiety symptoms.

### Methodological shift: From correlational studies to complex systems perspectives

CiteSpace burst analysis further reveals a significant shift in the methodological orientation of this field. In recent years, keywords such as “network analysis,” “epidemiology” and “resilience” have continuously burst, indicating that research on childhood adversity and anxiety is gradually shifting from univariate or linear association analysis to complex systems and multilevel modeling perspectives. This trend reflects the researchers’ attempts to break through the limitations of traditional latent variable models and understand the psychopathological process in a more dynamic and structured way. In this context, Borsboom et al.’s symptom network theory provides an important methodological foundation for conceptualizing mental disorders. This theory suggests that mental disorders are not driven by a single latent cause but arise from direct interactions between symptoms; when these interactions are strong enough, a symptom network can form a self-sustaining feedback structure, manifesting as a stable mental disorder state (Borsboom, 2017). Although symptom network theory has not yet developed into a fully mature unified theory of psychopathology, its perspective on the dynamic relationships between symptoms provides a new analytical framework for understanding the heterogeneity, resilience and susceptibility of anxiety disorders. On this basis, researchers have begun to incorporate childhood adversity into symptom network models, exploring its potential “bridging” or amplifying role between different symptoms, thus expanding the explanatory space of adversity research at the symptom level and viewing childhood adversity not only as a distal risk factor but as a key regulatory node in the network structure. Meanwhile, the burst emergence of biologically related keywords (such as DNA methylation, neural circuits and gene–environment interactions) suggests that the field is also advancing parallel explorations of biological mechanisms. Research such as Pollok et al.’s imaging meta-analysis shows a relatively consistent association between early life adversity and structural changes in brain regions such as the hippocampus, amygdala and anterior cingulate cortex, with age and adversity type-specific effects (Pollok et al., 2022). These studies provide important clues for understanding the neural basis of childhood adversity’s impact on anxiety and related emotional disorders. Additionally, Heim et al.’s systematic review of early life stress further points out that early adversity may have long-lasting effects on the emotional regulation system through sensitive period effects, gene–environment interactions and epigenetic mechanisms, thereby increasing the risk of mental disorders in adulthood. These findings reinforce the theoretical basis for viewing childhood adversity as a cross-level biological-psychological risk factor (Heim and Binder, 2012). However, despite the significant progress in network models and biological mechanism research, both remain relatively scattered in terms of time series, research design and theoretical integration. Compared with epidemiological and clinical association studies, a stable and unified multilevel integration framework has not yet been established. These results suggest that future research urgently needs to strengthen interdisciplinary collaboration based on complex systems approaches, systematically integrating symptom network structures, neurobiological mechanisms and evidence of social environmental exposures to deepen the overall understanding of the relationship between childhood adversity and anxiety.

### High-citation literature and core authors: Foundational contributions and focus

High-citation literature analysis shows that the theoretical frameworks and empirical foundations in childhood adversity and anxiety research have been shaped primarily by a few seminal studies. From a global citation perspective, high-impact articles are mainly concentrated on neurobiological modeling of anxiety and trauma-related disorders, and the systematic integration of childhood adversity as a broad risk factor for mental disorders, reflecting the early developmental stage of this field, focused on theoretical construction and methodological integration. In terms of neurobiological mechanisms, Etkin et al.’s functional neuroimaging meta-analysis ranks first in global citations, systematically integrating brain function abnormalities in PTSD, social anxiety disorder and specific phobias in emotional processing. This study revealed the common neurobiological basis of anxiety disorders and normal fear responses in the overactivation of the amygdala and insula, while pointing out some specificity in PTSD regarding functional reduction in the anterior cingulate cortex and ventromedial prefrontal cortex. This study, by synthesizing heterogeneous research results, provided a unified neuroimaging framework for the common and specific neurobiological mechanisms of anxiety disorders, laying the important groundwork for subsequent research. Following this, Shin et al.’s systematic review of the neural circuits of anxiety disorders further confirmed that the fear circuit model proposed in animal fear models and human emotional processing studies could explain the enhanced amygdala and insula responses in various anxiety disorders, while PTSD shows a characteristic reduction in the regulatory functions of the anterior cingulate cortex and ventromedial prefrontal cortex. These studies strengthened the amygdala–prefrontal cortex–hippocampus pathway hypothesis for anxiety disorder neural circuits and highlighted key issues such as whether functional abnormalities are risk susceptibility factors or disease consequences (Milad et al., 2009; Shin and Liberzon, 2010). Early imaging studies, such as Bremner et al.’s PET study on Vietnam War veterans, revealed functional abnormalities in areas such as the medial prefrontal cortex and anterior cingulate cortex in PTSD patients from the perspective of trauma cue processing, suggesting that brain region changes related to emotional regulation, memory processing and threat response are crucial neural bases for PTSD symptoms (Bremner et al., 1999). Overall, these high-citation studies surrounding fear circuits, extinction learning and trauma cue processing have collectively established the mainstream research paradigm for explaining anxiety and trauma-related disorders through neuroimaging and fear conditioning models, providing key theoretical and methodological support for integrating childhood adversity into neurobiological research on anxiety disorders.

Meanwhile, in the field of childhood adversity research, Chen et al.’s systematic review and meta-analysis integrated large-scale population evidence, systematically assessing the association between childhood sexual abuse and lifelong mental disorder diagnoses, clearly pointing out the robust relationship between childhood sexual abuse and the significantly increased risk of anxiety disorders, depression, PTSD and suicide attempts. This research provided an important evidence base for childhood adversity as a broad risk factor for mental disorders, marking the transition from fragmented association studies to systematic evidence integration in the field (Chen et al., 2010). Building on this, McLaughlin et al.’s “stress sensitization hypothesis” further explained how childhood adversity increases the risk of mental disorders by altering individual sensitivity to subsequent stress exposure, providing a theoretical framework for understanding the long-term association between childhood adversity and adult mental disorders. This theory has become an important theoretical anchor for linking epidemiological evidence with psychological and biological mechanisms in research. As the research focus shifted from single disorder risk to psychopathological structures themselves, Lamers et al. revealed the highly stable comorbidity pattern between anxiety and depression disorders in the NESDA cohort, noting that comorbidity was closely related to more childhood trauma experiences, higher susceptibility traits and more severe and persistent clinical manifestations (McLaughlin et al., 2010; Lamers et al., 2011). These studies highlight the limitations of traditional single-diagnosis frameworks in explaining complex real-world psychopathological structures and provide key empirical evidence for cross-diagnostic research. On this basis, Caspi et al.’s “p factor” model represents a significant milestone in the shift from categorical diagnosis to cross-diagnostic and dimensional models in psychopathology research. The model emphasizes that multiple mental disorders share the same risk factors and biological foundations, with early adverse experiences, such as childhood adversity, potentially influencing multiple disorder outcomes by increasing overall susceptibility to psychopathology (Caspi and Moffitt, 2018). The introduction of the “p factor” not only provides a theoretical explanation for the high comorbidity of anxiety, depression and other disorders but also further promotes the shift of childhood adversity research from disorder-specific analysis to cross-diagnostic risk mechanism integration. Overall, this series of high-citation studies outlines the clear research evolution from broad risk identification of childhood adversity to the systematic characterization of comorbidity structures and to the construction of cross-diagnostic and dimensional theoretical models. This highlights the limitations of single-diagnosis frameworks in explaining complex psychopathological structures and has had a profound impact on subsequent cross-diagnostic and mechanism integration research.

Compared to global high-citation literature, local citation analysis presents a more focused and consistent research orientation. In the literature sample of this study, the most cited articles primarily focus on the direct link between childhood adversity/abuse and anxiety disorders, particularly clinical and epidemiological evidence of anxiety disorder subtypes, such as panic disorder, social anxiety disorder and generalized anxiety disorder. This feature indicates that local high-citation literature plays a key role in solidifying the core empirical line and refining disorder-specific risk patterns. Safren et al.’s research, for example, systematically compared the prevalence of childhood physical or sexual abuse in different anxiety disorder diagnoses and found that childhood abuse was significantly higher in panic disorder patients compared to social anxiety disorder patients, with generalized anxiety disorder patients falling in between. Additionally, a history of childhood abuse was also associated with a higher risk of comorbid major depression in anxiety disorder patients. This type of study emphasizes that childhood adversity not only affects the onset risk of anxiety disorders but also closely relates to symptom severity and comorbidity structures. In the domain of social anxiety disorder, Simon et al.’s research further showed that childhood abuse had a higher prevalence among treatment-seeking generalized social anxiety disorder patients, with different types of abuse (especially emotional abuse and neglect) showing cumulative effects, correlating with more severe social anxiety symptoms and poorer functional outcomes. These findings highlight the important role of childhood adversity in the heterogeneity within anxiety disorder subtypes (Safren et al., 2002). Meanwhile, although Etkin et al.’s functional neuroimaging meta-analysis holds a core position in global citations, it also appears frequently in local citations, reflecting the important reference value of neuroimaging evidence in explaining childhood adversity-related anxiety symptoms (Etkin and Wager, 2007). These studies provide critical neurobiological insights into the commonalities and specificities of emotional processing and regulation circuits in different anxiety disorder subtypes. At the population level, research by Cougle, Goodwin and McLaughlin based on large-scale epidemiological data from the National Comorbidity Survey and others has systematically verified the independent association between childhood physical and sexual abuse and the risk of anxiety disorders in adulthood, and further proposed mechanisms such as stress sensitization to explain how childhood adversity increases the risk of anxiety and related mental disorders by enhancing individual sensitivity to subsequent stress events. The high citation frequency of these studies in local literature emphasizes their key role in bridging clinical observations with population-based evidence (Goodwin et al., 2005; Cougle et al., 2010; McLaughlin et al., 2010). Additionally, Spinhoven et al.’s analysis of the shared and specific effects of childhood adversity on anxiety and depression disorders represents an early manifestation of the cross-diagnostic perspective in local literature. However, their research still focuses on the differentiated relationship between types of adversity and specific disorder phenotypes (Spinhoven et al., 2010). Overall, global high-citation literature tends to reflect foundational contributions in theoretical modeling and the construction of cross-diagnostic frameworks, while local high-citation literature reveals a growing focus on more specific and stable patterns of association between childhood adversity and anxiety disorders. This difference suggests that, although the cross-diagnostic perspective is becoming increasingly important in psychopathological research, disorder-specific mechanisms related to anxiety disorder subtypes still hold irreplaceable research value in the study of childhood adversity and anxiety.”

### Insights and research frontiers

Overall, research on childhood adversity and anxiety has evolved from an early descriptive association phase to a mature research field that integrates multiple layers and methods. The bibliometric results show that this field has not only expanded in research topics from specific trauma types to cumulative adversity and cross-diagnostic perspectives but also shifted significantly in methodology from cross-sectional correlation analysis to longitudinal, mechanism-oriented and complex systems modeling. Based on the main findings of this study, future research can further deepen in the following directions. First, strengthening research on non-Western and underdeveloped regions is an important prerequisite for enhancing the generalizability and public health value of the field. Currently, high-output countries and influential institutions are still mainly concentrated in Europe and North America, with research samples having certain homogeneity in terms of sociocultural context, family structure, adversity types and coping resources. This structural bias may limit the applicability of research conclusions in different cultural and social contexts. Future studies should conduct more high-quality epidemiological and longitudinal research in regions such as Asia, Africa and Latin America, systematically comparing the manifestations, exposure patterns and impact pathways of childhood adversity on anxiety disorders across different cultural contexts, thereby constructing a more globally representative theoretical framework. Second, combining longitudinal study designs with multimodal data will help clarify the time series and potential causal pathways of childhood adversity’s impact on the occurrence and development of anxiety. Although a large body of research has confirmed the robust association between childhood adversity and anxiety risk, most of the evidence is still based on retrospective or cross-sectional designs, making it difficult to distinguish between risk susceptibility, disease consequences and mediating mechanisms. Future research could use prospective cohort designs, integrating psychological measurements, neuroimaging, physiological indicators and molecular biology data, to dynamically track individuals’ psychological and biological changes from childhood to adulthood, providing a more detailed understanding of critical windows for adversity exposure, cumulative effects and their influence on anxiety symptom trajectories. Third, further integrating network analysis, psychological resilience models and biological mechanism research will provide new opportunities for translating risk identification into intervention targets. The rise of symptom network models in recent years has allowed researchers to understand the internal structure and dynamic interactions of anxiety disorders at the symptom level, while resilience research provides an important perspective for explaining why individuals show different outcomes under similar adversity exposure. Future research should incorporate resilience-related variables into the network analysis framework, along with biological evidence, such as neural circuits, epigenetics and gene–environment interactions, to identify the “nodes” or “bridging mechanisms” that play a key regulatory role in the childhood adversity–anxiety pathway. This integrated approach will not only deepen understanding of the heterogeneity of anxiety disorders but also lay the foundation for the development of precise prevention and individualized intervention strategies. Overall, the bibliometric analysis of this study reveals the systematic evolution of childhood adversity and anxiety research in terms of themes, methods and theories. Future research, if breakthroughs are made in cross-cultural sample expansion, longitudinal causal mechanism analysis and multilevel integration models, is expected to drive the field from risk association descriptions to mechanism explanation and intervention translation, providing a more solid scientific foundation for the early identification and prevention strategies for anxiety disorders.

### Limitations

This study still has several limitations that should be interpreted with caution. First, this study employed a bibliometric method, and the analysis was limited to published literature, which is influenced by publication bias to some extent. Unpublished research, gray literature and non-English literature were not systematically included, which may lead to biased estimates of the research scale, topic distribution and impact. Second, although this study integrated three major databases (Web of Science Core Collection, Scopus and PubMed), it still could not fully cover all relevant research, especially regional journals or studies published in local languages. Therefore, the results of this study mainly reflect the research landscape under the international mainstream academic discourse system, rather than the global picture. Third, bibliometric analysis was based primarily on titles, abstracts, keywords and citation information, which cannot deeply assess the heterogeneity in research design, sample sources, childhood adversity measurement tools, anxiety disorder diagnostic criteria and statistical methods in the included studies. Therefore, the research hotspots and evolutionary trends presented in this study reflect changes in academic focus, rather than the strength of specific effect sizes or causal relationships. Fourth, this study is a cross-sectional bibliometric analysis and cannot directly infer causal pathways or temporal sequences between childhood adversity and anxiety disorders. Although some highly cited studies based on longitudinal cohort or mechanistic research have proposed theoretical hypotheses, this study itself did not reanalyze the original data, and its conclusions should be understood as descriptive and comparative evidence. Fifth, differences in the literature inclusion standards, keyword indexing rules and citation statistics between different databases may affect the publication volume, citation frequency and collaboration network structure. Although this study minimized such biases through deduplication and standardization, the impact could not be completely eliminated. Finally, although tools like CiteSpace and VOSviewer provide effective means to reveal research structures and theme evolution, their results still rely on parameter settings and algorithm assumptions, and different analytical strategies may lead to variations in the number of clusters and theme boundaries. Therefore, the related visualization results should be regarded as auxiliary interpretations of research trends rather than a strict categorization of research themes.

## Conclusion

This study conducted a bibliometric analysis of the research on childhood adversity and anxiety disorders based on the Web of Science Core Collection, Scopus and PubMed databases, providing a comprehensive overview of the development trajectory, academic structure and research frontiers of this field over the past few decades. The results show that research on childhood adversity and anxiety has undergone a significant shift from early scattered exploration to rapid growth over the past two decades, gradually forming a research system supported by interdisciplinary fields such as psychiatry, psychology, public health and neuroscience.

In terms of research theme evolution, the field initially focused on the relationship between specific trauma types and particular anxiety disorder diagnoses. It then gradually shifted toward the framework of cumulative adversity centered around adverse childhood experiences (ACEs), and in the past decade, it has significantly expanded to integrate cross-diagnostic risk factors, emotional regulation, psychological resilience and symptom networks. This evolutionary process reflects the limitations of the traditional single-diagnosis paradigm in explaining complex psychopathological structures and highlights the shift from a “exposure-disease” linear association to multilevel, dynamic mechanism models in research paradigms.

In terms of academic structure, the United States and European countries dominate in publication volume, citation frequency and core research institutions, forming the major knowledge production centers in the field. Meanwhile, while research output in regions such as Asia is on the rise, its overall international influence still needs to be enhanced. High-citation literature analysis further reveals that global high-impact research primarily focuses on neurobiological mechanism modeling, cross-diagnostic theoretical frameworks and the systematic integration of evidence, while local high-citation literature is more focused on the stable and specific clinical and epidemiological associations between childhood adversity and anxiety disorder subtypes. These two aspects complement each other in theory-building and empirical consolidation.

Methodologically, keyword burst and clustering analyses show that the field is gradually incorporating symptom network analysis, multimodal longitudinal design and biological mechanism research, marking a shift in research focus from risk identification to mechanism explanation and exploration of potential intervention targets. Although relevant studies have not yet formed a unified multi-level integration model, existing evidence has provided important insights into how childhood adversity affects the onset and maintenance of anxiety symptoms through psychological, neurobiological and social pathways. Overall, this study systematically reveals the evolution of research on childhood adversity and anxiety in terms of themes, methods and theories, offering researchers a clear overall picture of the knowledge structure and development direction of this field. Future research, if it can make further breakthroughs in expanding cross-cultural samples, longitudinal causal mechanism analysis and complex system model integration, is expected to drive the field from descriptive associations toward mechanism integration and precision interventions, providing a more solid scientific foundation for the early identification and prevention strategies of anxiety disorders.

## Supporting information

10.1017/gmh.2026.10235.sm001 Ruan et al. supplementary material Ruan et al. supplementary material

## Data Availability

The data supporting the findings of this study were derived from publicly available literature databases, including Web of Science Core Collection, Scopus and PubMed. All data used in this study were obtained from published records, and the search strategy, inclusion criteria and data processing procedures are described in the Methods section. Further details are available from the corresponding author upon reasonable request.

## Supplementary material

The supplementary material for this article can be found at http://doi.org/10.1017/gmh.2026.10235.

## Long descriptions

### Figure 1. Long description

The flowchart consists of three levels.

At the top level, three blue boxes represent the initial search results from different databases.

1. Web of Science Core Collection: Search strategy includes Anxiety disorders AND early-life adversity forward slash A C E forward slash childhood maltreatment T S. N equals 1034.

2. Scopus: Search strategy includes Anxiety disorders AND early-life adversity forward slash A C E forward slash childhood maltreatment T I T L E minus A B S minus K E Y. N equals 4068.

3. PubMed: Search strategy includes Anxiety disorders AND early-life adversity forward slash A C E forward slash childhood maltreatment Title or Abstract. N equals 689.

Arrows from these three boxes converge and point down to a central red box labeled Exclusion. The exclusion criteria are listed as: 1. Studies unrelated to Anxiety disorders and A C E. 2. Non-academic articles such as conference abstracts, book reviews, or editorials. 3. Duplicate publications.

An arrow points from the exclusion box down to a final yellow box at the bottom. This box states: Merge document data from Web of Science, Scopus, and Pubmed using the bibliometrix package. Duplicate documents removed, resulting in 4481 articles.

Navigate back to Figure 1..

### Figure 2. Long description

The x-axis lists years from 1972 to 2026 in three-year increments. The y-axis measures publication count from 0 to 400. The data is visualized as a series of light green, pointed vertical peaks.

* From 1972 to 1989, publication counts are extremely low, ranging from 0 to 2.

* From 1990 to 2000, a slow increase begins, with counts rising from 4 to 25.

* From 2001 to 2010, the growth accelerates, reaching 75 publications.

* From 2011 to 2025, the chart shows an exponential upward curve. Key data points include 109 in 2011, 175 in 2017, 279 in 2021, and peaking at 416 in 2025.

* The final data point for 2026 shows a sharp decline to 24, likely representing incomplete data for the current or future year.

Navigate back to Figure 2..

### Table 1. Long description

The table contains six columns: Country, Articles, Articles percent, S C P (Single Country Publications), M C P (Multiple Country Publications), and M C P percent. The data for the top 10 countries is as follows:

* U S A: 1,385 articles (30.9 percent), 1,327 S C P, 58 M C P, 4.2 percent M C P.

* CANADA: 294 articles (6.6 percent), 271 S C P, 23 M C P, 7.8 percent M C P.

* CHINA: 251 articles (5.6 percent), 223 S C P, 28 M C P, 11.2 percent M C P.

* UNITED KINGDOM: 226 articles (5 percent), 210 S C P, 16 M C P, 7.1 percent M C P.

* GERMANY: 221 articles (4.9 percent), 194 S C P, 27 M C P, 12.2 percent M C P.

* NETHERLANDS: 188 articles (4.2 percent), 173 S C P, 15 M C P, 8 percent M C P.

* AUSTRALIA: 180 articles (4 percent), 163 S C P, 17 M C P, 9.4 percent M C P.

* ITALY: 108 articles (2.4 percent), 101 S C P, 7 M C P, 6.5 percent M C P.

* FRANCE: 79 articles (1.8 percent), 69 S C P, 10 M C P, 12.7 percent M C P.

* BRAZIL: 78 articles (1.7 percent), 62 S C P, 16 M C P, 20.5 percent M C P.

Navigate back to Table 1..

### Figure 3. Long description

The figure consists of three horizontal bar charts labeled A, B, and C.

Panel A displays the top 10 journals. The x-axis represents publication count from 0 to 240. From highest to lowest:

- JOURNAL OF AFFECTIVE DISORDERS: 232

- CHILD ABUSE AND NEGLECT: 140

- FRONTIERS IN PSYCHIATRY: 106

- JOURNAL OF PSYCHIATRIC RESEARCH: 102

- PSYCHIATRY RESEARCH: 83

- PSYCHOLOGICAL MEDICINE: 64

- DEPRESSION AND ANXIETY: 58

- B M C PSYCHIATRY: 56

- PLOS ONE: 56

- JOURNAL OF NERVOUS AND MENTAL DISEASE: 48

Panel B displays the top 10 institutions. The x-axis represents publication count from 0 to 200. From highest to lowest:

- HARVARD MEDICAL SCHOOL: 193

- UNIVERSITY OF TORONTO: 165

- HARVARD UNIVERSITY: 155

- KING’S COLLEGE LONDON: 154

- UNIVERSITY OF CALIFORNIA: 139

- VRIJE UNIVERSITEIT AMSTERDAM: 101

- UNIVERSITY OF MANITOBA: 91

- COLUMBIA UNIVERSITY: 88

- YALE SCHOOL OF MEDICINE: 87

- MCMASTER UNIVERSITY: 80

Panel C displays the top 10 authors. The x-axis represents publication count from 0 to 65. From highest to lowest:

- PENNINX B: 65

- LEE S: 44

- WANG Y: 43

- ZHANG Y: 41

- KESSLER R: 37

- LI Y: 33

- NEMEROFF C: 30

- STEIN D: 30

- WANG J: 28

- CHEN Y: 27

Navigate back to Figure 3..

### Figure 4. Long description

The diagram is a circular Voronoi treemap where the area of each polygonal cell corresponds to the frequency count of a specific keyword.

At the center is the core keyword:

* article: 2686

Surrounding the center are several large primary clusters:

* human: 5402 (the largest cell, located in the bottom-left quadrant)

* female: 2677 (top-center)

* anxiety disorder: 2461 (top-right)

* depression: 2360 (middle-left)

* male: 2283 (bottom-center)

* adult: 2207 (middle-left)

Intermediate-sized cells clustered around the center include:

* major clinical study: 1581

* anxiety: 1438

* controlled study: 1323

* adolescent: 1287

* child: 1260

* posttraumatic stres…: 1071

* child abuse: 1042

* sexual abuse: 1005

Smaller peripheral cells include:

* psychology: 1056

* mental disease: 954

* prevalence: 868

* anxiety disorders: 855

* mental health: 852

* priority journal: 832

* physical abuse: 820

* risk factor: 821

* young adult: 727

* middle aged: 729

* comorbidity: 711

* questionnaire: 703

* major depres…: 680

* emotional abuse: 625

* childhood trau…: 620

Navigate back to Figure 4..

### Figure 5. Long description

Panel A is a keyword co-occurrence network with a dense central core and a lighter perimeter. At the center, the largest nodes represent depression, generalized anxiety disorder, and posttraumatic-stress-disorder. Surrounding this core are secondary nodes including adverse childhood experiences, prevalence, and panic disorder. The perimeter contains specialized terms like D N A methylation, serotonin transporter gene, and covid-19. A color scale at the bottom right indicates a timeline from 2014 in dark blue to 2020 in bright yellow.

Panel B is an institutional collaboration network organized into colored clusters. The red and orange cluster in the center-left features Columbia Univ, Harvard Med Sch, and Stanford Univ. The blue cluster in the center-right includes Univ Groningen and Leiden Univ. The green cluster on the far left contains Beijing Normal Univ and Capital Med Univ. The purple cluster at the bottom includes Univ Toronto and McMaster Univ. The light blue cluster on the far right features Univ Munster and Heidelberg Univ. Lines of varying thickness connect these nodes to indicate the strength of co-authorship ties.

Navigate back to Figure 5..

### Figure 6. Long description

Panel A is a radial network diagram generated by CiteSpace. At the center is cluster number 0 maternal separation. Surrounding this core are several interconnected clusters: number 1 stressful life event at the top, number 2 panic disorder and number 3 adverse childhood experience to the right, number 4 non-psychotic disorder and number 5 non-suicidal self-injury at the bottom, and number 6 mood disorder and number 7 network analysis to the left. Each cluster contains numerous smaller red-labeled keywords such as depressive symptoms, cognitive behavioral therapy, and D S M-I V.

Panel B is a table titled Top 24 Keywords with the Strongest Citation Bursts. The table has columns for Keywords, Year, Strength, Begin, End, and a timeline from 2016 to 2026.

* The top three keywords by strength are women (4.79), sexual-abuse (4.28), and dsm-iv (4.11).

* Keywords with the earliest bursts (2016) include sexual-abuse, early life stress, and panic disorder.

* The most recent bursts (starting in 2023) include scale, network analysis, epidemiology, and resilience, with scale and network analysis projected to continue through 2026.

* The timeline column uses red horizontal bars to indicate the duration of the citation burst against a light blue background representing the total study period.

Navigate back to Figure 6..

### Figure 7. Long description

A two-panel vertical layout.

Panel A, at the top, is a lollipop chart titled A. The y-axis represents citation counts from 0 to 30,000 in increments of 2,000. The x-axis lists 10 countries. From left to right, the data points are:

- U S A: 25,523 citations (large purple circle)

- G E R M A N Y: 3,611 citations (pink circle)

- C A N A D A: 2,946 citations (yellow circle)

- N E T H E R L A N D S: 2,938 citations (light blue circle)

- A U S T R A L I A: 1,723 citations (dark grey circle)

- C H I N A: 1,265 citations (blue circle)

- U N I T E D K I N G D O M: 1,201 citations (green circle)

- B R A Z I L: 896 citations (red-pink circle)

- I T A L Y: 762 citations (orange circle)

- S O U T H A F R I C A: 658 citations (brown circle)

Panel B, at the bottom, is a lollipop chart titled B. The y-axis represents citation counts from 0 to 2,600 in increments of 200. The x-axis lists 10 journals. From left to right, the data points are:

- A M J P S Y C H I A T: 2,250 citations (purple)

- J A F F E C T D I S O R D E R S: 1,764 citations (pink)

- A R C H G E N P S Y C H I A T: 1,755 citations (yellow)

- B I O L P S Y C H I A T: 1,425 citations (light blue)

- C H I L D A B U S E N E G L E C T: 1,410 citations (dark grey)

- P S Y C H O L M E D: 1,350 citations (blue)

- D E P R E S S A N X I E T Y: 901 citations (green)

- B R I T J P S Y C H I A T: 795 citations (red-pink)

- P S Y C H I A T R E S: 736 citations (orange)

- J C L I N P S Y C H I A T: 734 citations (brown)

Navigate back to Figure 7..

### Figure 8. Long description

The y-axis represents citation counts ranging from 0 to 120 in increments of 10. The x-axis lists author names. The data is presented as colored circles atop vertical lines, with the citation count written above each circle.

From left to right, the authors and their citation counts are:

* STEIN M B: 105 citations (large purple circle)

* POLLACK M H: 70 citations (pink circle)

* DOMSCHKE K: 69 citations (yellow circle)

* PENNINX B W J H: 66 citations (light blue circle)

* DECKERT J: 60 citations (dark grey circle)

* SPINHOVEN P: 60 citations (medium blue circle)

* PAULI P: 57 citations (green circle)

* REIF A: 57 citations (reddish-pink circle)

* WALKER J R: 52 citations (orange circle)

* BANDELOW B: 48 citations (brown circle)

* BROOCKS A: 48 citations (light purple circle)

* HAJAK G: 48 citations (blue-purple circle)

* MANCINI C: 48 citations (dark blue circle)

* RÜTHER E: 48 citations (teal circle).

Navigate back to Figure 8..

### Table 2. Long description

The table contains six columns: Number, First author, Article name, Source, Year, and Global citations.

* Rank 1: Amit Etkin. Functional neuroimaging of anxiety: a meta-analysis of emotional processing in P T S D, social anxiety disorder, and specific phobia. Am J Psychiatry, 2007. 2,521 citations.

* Rank 2: Lisa M Shin. The neurocircuitry of fear, stress, and anxiety disorders. Neuropsychopharmacology, 2010. 1,486 citations.

* Rank 3: Mohammed R Milad. Neurobiological basis of failure to recall extinction memory in posttraumatic stress disorder. Biol Psychiatry, 2009. 1,039 citations.

* Rank 4: Laura P Chen. Sexual abuse and lifetime diagnosis of psychiatric disorders: systematic review and meta-analysis. Mayo Clin Proc, 2010. 730 citations.

* Rank 5: Femke Lamers. Comorbidity patterns of anxiety and depressive disorders in a large cohort study: the Netherlands Study of Depression and Anxiety (N E S D A). J Clin Psychiatry, 2011. 693 citations.

* Rank 6: M C Zanarini. Axis I comorbidity of borderline personality disorder. Am J Psychiatry, 1998. 673 citations.

* Rank 7: Avshalom Caspi. All for One and One for All: Mental Disorders in One Dimension. Am J Psychiatry, 2018. 665 citations.

* Rank 8: K A McLaughlin. Childhood adversity, adult stressful life events, and risk of past-year psychiatric disorder: a test of the stress sensitization hypothesis in a population-based sample of adults. Psychol Med, 2010. 643 citations.

* Rank 9: J D Bremner. Neural correlates of exposure to traumatic pictures and sound in Vietnam combat veterans with and without posttraumatic stress disorder: a positron emission tomography study. Biol Psychiatry, 1999. 513 citations.

* Rank 10: Charles B Nemeroff. Posttraumatic stress disorder: a state-of-the-science review. J Psychiatr Res, 2006. 478 citations.

Navigate back to Table 2..

### Table 3. Long description

The table contains six columns: Number, First author, Article name, Source, Year, and Local citations.

* Rank 1: Steven A Safren, History of childhood abuse in panic disorder, social phobia, and generalized anxiety disorder, J Nerv Ment Dis, 2002, 35 citations.

* Rank 2: Amit Etkin, Functional neuroimaging of anxiety: a meta-analysis of emotional processing in P T S D, social anxiety disorder, and specific phobia, Am J Psychiatry, 2007, 35 citations.

* Rank 3: Naomi M Simon, Childhood maltreatment linked to greater symptom severity and poorer quality of life and function in social anxiety disorder, Depress Anxiety, 2009, 35 citations.

* Rank 4: Borwin Bandelow, Early traumatic life events, parental rearing styles, family history of mental disorders, and birth risk factors in patients with social anxiety disorder, Eur Arch Psychiatry Clin Neurosci, 2004, 27 citations.

* Rank 5: Jesse R Cougle, Examining the unique relationships between anxiety disorders and childhood physical and sexual abuse in the National Comorbidity Survey-Replication, Psychiatry Res, 2010, 27 citations.

* Rank 6: Renee D Goodwin, Childhood abuse and familial violence and the risk of panic attacks and panic disorder in young adulthood, Psychol Med, 2005, 26 citations.

* Rank 7: Laura P Chen, Sexual abuse and lifetime diagnosis of psychiatric disorders: systematic review and meta-analysis, Mayo Clin Proc, 2010, 24 citations.

* Rank 8: K A McLaughlin, Childhood adversity, adult stressful life events, and risk of past-year psychiatric disorder: a test of the stress sensitization hypothesis in a population-based sample of adults, Psychol Med, 2010, 24 citations.

* Rank 9: Philip Spinhoven, The specificity of childhood adversities and negative life events across the life span to anxiety and depressive disorders, J Affect Disord, 2010, 23 citations.

* Rank 10: Borwin Bandelow, Early traumatic life events, parental attitudes, family history, and birth risk factors in patients with panic disorder, Compr Psychiatry, 2002, 21 citations.

Navigate back to Table 3..
