# Supplementary material for: Bibliometric analysis of childhood adversity and anxiety disorders: Trends, hotspots and international collaboration
Source: Glob Ment Health (Camb). 2026 Jun 8;13:e153. doi: 10.1017/gmh.2026.10235 (PMC13375840; doi:10.1017/gmh.2026.10235)
Supplement: Ruan et al. supplementary material [file S2054425126102350sup001.docx]

Table S1 Detailed search strategies for each database

| **Database** | **Search field** | **Search strategy** |
| --- | --- | --- |
| WoSCC | TS | TS=("anxiety disorder" OR "separation anxiety disorder" OR "selective mutism" OR "specific phobia" OR "isolated phobia" OR "social anxiety disorder" OR "social phobias" OR "panic disorder" OR "episodic paroxysmal anxiety" OR "agoraphobia" OR "generalized anxiety disorder" OR "phobic anxiety disorder" OR "mixed anxiety disorders")  AND  TS=("early life stress events" OR "early life stress" OR "early adversity" OR "childhood trauma" OR "stress in early life" OR "adverse childhood experiences" OR "adversity in early life" OR "childhood maltreatment" OR "childhood abuse" OR "childhood stress" OR "Early Adverse Life Events" OR "Childhood adversity" OR "emotional abuse" OR "emotional maltreatment" OR "physical abuse" OR "physical maltreatment" OR "severe physical abuse" OR "bodily maltreatment" OR "sexual maltreatment" OR "sexual abuse" OR "sex abuse" OR "ELA" OR "early life adversity" OR "adverse childhood") |
| Scopus | TITLE-ABS-KEY | TITLE-ABS-KEY("anxiety disorder" OR "separation anxiety disorder" OR "selective mutism" OR "specific phobia" OR "isolated phobia" OR "social anxiety disorder" OR "social phobias" OR "panic disorder" OR "episodic paroxysmal anxiety" OR "agoraphobia" OR "generalized anxiety disorder" OR "phobic anxiety disorder" OR "mixed anxiety disorders")  AND  TITLE-ABS-KEY("early life stress events" OR "early life stress" OR "early adversity" OR "childhood trauma" OR "stress in early life" OR "adverse childhood experiences" OR "adversity in early life" OR "childhood maltreatment" OR "childhood abuse" OR "childhood stress" OR "Early Adverse Life Events" OR "Childhood adversity" OR "emotional abuse" OR "emotional maltreatment" OR "physical abuse" OR "physical maltreatment" OR "severe physical abuse" OR "bodily maltreatment" OR "sexual maltreatment" OR "sexual abuse" OR "sex abuse" OR "ELA" OR "early life adversity" OR "adverse childhood") |
| PubMed | Title/Abstract | A("anxiety disorder" OR "separation anxiety disorder" OR "selective mutism" OR "specific phobia" OR "isolated phobia" OR "social anxiety disorder" OR "social phobia" OR "panic disorder" OR "episodic paroxysmal anxiety" OR "agoraphobia" OR "generalized anxiety disorder" OR "phobic anxiety disorder" OR "mixed anxiety disorders")  AND  ("early life stress events" OR "early life stress" OR "early adversity" OR "childhood trauma" OR "stress in early life" OR "adverse childhood experiences" OR "adversity in early life" OR "childhood maltreatment" OR "childhood abuse" OR "childhood trauma" OR "childhood stress" OR "Early Adverse Life Events" OR "Childhood adversity" OR "emotional abuse" OR "emotional maltreatment" OR "physical abuse" OR "physical maltreatment" OR "severe physical abuse" OR "bodily maltreatment" OR "sexual maltreatment" OR "sexual abuse" OR "sex abuse" OR "ELA" OR "early life adversity" OR "adverse childhood") |
