# Supplementary material for: Bibliometric analysis of childhood adversity and anxiety disorders: Trends, hotspots and international collaboration
Source: Glob Ment Health (Camb). 2026 Jun 8;13:e153. doi: 10.1017/gmh.2026.10235 (PMC13375840; doi:10.1017/gmh.2026.10235)
Supplement: Peer review history

# Reviewer report: Review: Bibliometric analysis of childhood adversity and anxiety disorders: Trends, hotspots and international collaboration — R0/PR2

**DOI:** 10.1017/gmh.2026.10235.pr2

The introduction provides a good overview of childhood adversity and anxiety research; however, the authors should clarify the specific research gap addressed by this bibliometric study. Previous bibliometric analyses have explored trauma, adverse childhood experiences, and mental health trends. The manuscript should clearly state how this study extends or differs from previous bibliometric research.

What specific gap in bibliometric literature does this study address?

Whether previous bibliometric analyses have examined ACE–mental health or trauma–psychiatry research.

How does this analysis provide new insights beyond existing bibliometric work?

Reduce narrative literature review sections and focus more on interpreting the bibliometric findings.

The study includes three databases: WoSCC, Scopus, and PubMed, which is appropriate for interdisciplinary research. However, the authors should further justify:

Why Google Scholar, Dimensions, or PsycINFO were not included.

Potential database coverage overlap between Scopus and Web of Science.

How duplicate records across databases were handled.

The terms childhood adversity, early life adversity, and adverse childhood experiences (ACE) are used interchangeably. The manuscript should clarify the conceptual distinctions among these terms.

The manuscript addresses an important research topic and provides valuable insights into the development of childhood adversity and anxiety research. With improvements in methodological transparency, theoretical integration, and clarity of presentation, the paper will make a meaningful contribution to the literature on global mental health and developmental psychopathology.

# Reviewer report: Review: Bibliometric analysis of childhood adversity and anxiety disorders: Trends, hotspots and international collaboration — R0/PR3

**DOI:** 10.1017/gmh.2026.10235.pr3

**Authors:** Kalpana Purushothaman

**Affiliations:** Independent Scholar, India

“The linkage between childhood adversities and anxiety outcomes is articulated well and the results are presented in a clear and logically structured manner, making the findings accessible to both academic and applied audiences. It may be valuable to further situate the findings within diverse socio-cultural contexts, particularly given how childhood adversity is experienced and interpreted differently across settings.

# Editor report: Recommendation: Bibliometric analysis of childhood adversity and anxiety disorders: Trends, hotspots and international collaboration — R0/PR4

**DOI:** 10.1017/gmh.2026.10235.pr4

**Affiliations:** Department of Primary Care & Mental Health, University of Liverpool, UK, United Kingdom of Great Britain and Northern Ireland

Dear Authors 

We have now received comments form reviewers. In light of the comments, we recommend minor revisions to the current manuscript, for us to be able to consider it for publication in the Journal. 

Regards

Siham

# Editor report: Decision: Bibliometric analysis of childhood adversity and anxiety disorders: Trends, hotspots and international collaboration — R0/PR5

**DOI:** 10.1017/gmh.2026.10235.pr5

**Affiliations:** London School of Hygiene & Tropical Medicine, United Kingdom of Great Britain and Northern Ireland

No accompanying comment.

# Editor report: Recommendation: Bibliometric analysis of childhood adversity and anxiety disorders: Trends, hotspots and international collaboration — R1/PR7

**DOI:** 10.1017/gmh.2026.10235.pr7

**Affiliations:** Department of Primary Care & Mental Health, University of Liverpool, UK, United Kingdom of Great Britain and Northern Ireland

Dear Authors 

Thanks for submitting the revised version of the manuscript. We have reviewed the manuscript and it is accepted for publication. Congratulations! 

We will inform you about the next steps. 

Warmly 

Siham

# Editor report: Decision: Bibliometric analysis of childhood adversity and anxiety disorders: Trends, hotspots and international collaboration — R1/PR8

**DOI:** 10.1017/gmh.2026.10235.pr8

**Affiliations:** London School of Hygiene & Tropical Medicine, United Kingdom of Great Britain and Northern Ireland

No accompanying comment.
